# Implicit and explicit iterative algorithms for hierarchical variational inequality in uniformly smooth Banach spaces

**DOI:** 10.1186/s13660-017-1523-8

**Published:** 2017-10-04

**Authors:** Lu-Chuan Ceng, Yung-Yih Lur, Ching-Feng Wen

**Affiliations:** 1 Department of Mathematics, Shanghai Normal University, Shanghai, 200234 China; 2 Department of Industrial Management, Vanung University, Taoyuan, Taiwan; 3 Center for Fundamental Science; and Research Center for Nonlinear Analysis and Optimization, Kaohsiung Medical University, Kaohsiung, 80702 Taiwan; 4 Department of Medical Research, Kaohsiung Medical University Hospital, Kaohsiung, 80702 Taiwan

**Keywords:** 49J30, 47H09, 47J20, 49M05, system of variational inequalities, hierarchical variational inequality, nonexpansive mapping, fixed point, implicit and explicit iterative algorithms, global convergence

## Abstract

The purpose of this paper is to solve the hierarchical variational inequality with the constraint of a general system of variational inequalities in a uniformly convex and 2-uniformly smooth Banach space. We introduce implicit and explicit iterative algorithms which converge strongly to a unique solution of the hierarchical variational inequality problem. Our results improve and extend the corresponding results announced by some authors.

## Introduction

Let *X* be a real Banach space with its topological dual $X^{*}$, and *C* be a nonempty closed convex subset of *X*. Let $T:C\to X$ be a nonlinear mapping on *C*. We denote by $\operatorname{Fix}(T)$ the set of fixed points of *T* and by **R** the set of all real numbers. A mapping $T:C\to X$ is called *L*-Lipschitz continuous if there exists a constant $L\geq0$ such that

$$\Vert Tx-Ty \Vert \leq L \Vert x-y \Vert ,\quad\forall x,y\in C. $$

In particular, if $L=1$ then *T* is called a nonexpansive mapping; if $L\in[0,1)$ then *T* is called a contraction.

The normalized dual mapping $J:X\to2^{X^{*}}$ is defined as

1 $$ J(x):=\bigl\{ \varphi\in X^{*}:\langle x,\varphi\rangle= \Vert x \Vert ^{2}= \Vert \varphi \Vert ^{2}\bigr\} ,\quad \forall x\in X, $$

where $\langle\cdot,\cdot\rangle$ denotes the generalized duality pairing; see, e.g., [1] for further details.

Let $U:=\{x\in X: \Vert x \Vert =1\}$ be the unit sphere of *X*. Then the space *X* is said to

- have a Gâteaux differentiable norm if the limit $\lim_{t\to0^{+}}( \Vert x+ty \Vert - \Vert x \Vert )/t$ exists for each $x,y\in U$.
- have a uniformly Gâteaux differentiable norm if the limit is attained uniformly for $x\in U$.
- be strictly convex if and only if for $x,y\in U$ with $x\neq y$, we have
  $$\bigl\Vert (1-\lambda)x+\lambda y \bigr\Vert < 1,\quad \forall\lambda\in(0,1). $$

We note that if *X* is smooth, then the normalized duality mapping is single-valued; and if the norm of *X* is uniformly Gâteaux differentiable, then the normalized duality mapping is norm to weak star uniformly continuous on every bounded subset of *X* (see [1]). In the sequel, we shall denote by *j* the single-valued normalized duality mapping.

Let *X* be a smooth Banach space. Let $A,B:C\to X$ be two nonlinear mappings and $\lambda,\mu$ be two positive real numbers. The general system of variational inequalities (GSVI, for short) is to find $(x^{*},y^{*})\in C\times C$ such that

2 $$ \textstyle\begin{cases} \langle\lambda Ay^{*}+x^{*}-y^{*},j(x-x^{*})\rangle\geq0,& \forall x\in C,\\ \langle\mu Bx^{*}+y^{*}-x^{*},j(x-y^{*})\rangle\geq0,&\forall x\in C. \end{cases} $$

The equivalence between GSVI (2) and the fixed point problem of some nonexpansive mapping defined on a Banach space is established in Yao et al. [2]. The authors [2] introduced and analyzed implicit and explicit iterative algorithms for solving GSVI (2) by using this equivalence, and they proved the strong convergence of the sequences generated by the proposed algorithms. Subsequently, Ceng et al. [3] proposed and analyzed an implicit algorithm of Mann’s type and another explicit algorithm of Mann’s type for solving GSVI (2).

If *X* is a real Hilbert space, then GSVI (2) was introduced and studied by Ceng et al. [4]. In this case, for $A=B$, it was considered by Verma [5] (see also [6]). Further, in this case, when $x^{*}=y^{*}$, problem (2) reduces to the following classical variational inequality (in short, VI) of finding $x^{*}\in C$ such that

3 $$ \bigl\langle Ax^{*},x-x^{*}\bigr\rangle \geq0,\quad\forall x\in C. $$

This problem is a fundamental problem in the variational analysis, in particular, in the optimization theory and mechanics; see, e.g., [7–12] and the references therein. A large number of algorithms for solving this problem are essentially projection algorithms that employ projections onto the feasible set *C* of the VI, or onto some related set, so as to iteratively reach a solution. In particular, Korpelevich [13] proposed extragradient method for solving the VI in the Euclidean space. This method further has been improved by several researchers; see, e.g., [4, 14, 15] and the references therein.

In the case of a Banach space setting, that is, if $A=B$ and $x^{*}=y^{*}$, the VI is defined as

4 $$ \bigl\langle Ax^{*},j\bigl(x-x^{*}\bigr)\bigr\rangle \geq0,\quad\forall x\in C. $$

Aoyama et al. [16] proposed an iterative scheme to find the approximate solution of (4) and proved the weak convergence of the sequences generated by the proposed scheme. It is also well known [16] that, in a smooth Banach space, this problem is equivalent to a fixed-point equation, containing a sunny nonexpansive retraction from any point of the space onto the feasible set, which is usually assumed to be closed and convex. For the complexity of the feasible set, the sunny nonexpansive retraction is difficult to compute. To overcome this drawback in a Hilbert space, where the retraction is a metric projection, in Yamada [17], the feasible set is assumed to be the common fixed points of a finite family of nonexpansive mappings, and an explicit hybrid steepest-descent method is introduced. In this case, the variational inequality defined on such a feasible set is also called a hierarchical variational inequality (in short, HVI). Yamada’s method is then extended to solve more complex problems involving finite or infinite nonexpansive mappings (one can refer to, e.g., [18, 19] and the references therein). Zeng and Yao [19] introduced an implicit method that converges weakly to a solution of a variational inequality, containing a Lipschitz continuous and strongly monotone mapping in a Hilbert space *H*, where the feasible set is the common fixed points of a finite family of nonexpansive mappings on *H*. Ceng et al. [20] extended this result from nonexpansive mappings to Lipschitz pseudocontractive mappings and strictly pseudocontractive mappings on *H*. Recently, Buong and Anh [21] modified Yamada’s result and proposed a strongly convergent implicit method.

In this paper, we are going to solve the hierarchical variational inequality with the constraint of a general system of variational inequalities in a uniformly convex and 2-uniformly smooth Banach space. We introduce implicit and explicit iterative algorithms for finding a solution of the problem and derive the strong convergence of the proposed algorithms to a unique solution of the problem. Our results improve and extend the corresponding results announced by some others, e.g., Ceng et al. [3] and Buong and Phuong [18].

## Preliminaries

Let *X* be a real Banach space with the dual space $X^{*}$. For simplicity, the norms of *X* and $X^{*}$ are denoted by the symbol $\| \cdot\|$. Let *X* be a nonempty closed convex subset of a real Banach space *X*. We write $x_{n}\rightharpoonup x$ (respectively, $x_{n}\to x$) to indicate that the sequence $\{x_{n}\}$ converges weakly (respectively, strongly) to *x*. A mapping $J:X\to2^{X^{*}}$, defined by

$$J(x)=\bigl\{ \varphi\in X^{*}:\langle x,\varphi\rangle= \Vert \varphi \Vert ^{2}\mbox{ and } \Vert \varphi \Vert = \Vert x \Vert \bigr\} , $$

is called the normalized duality mapping of *X*. We know that $J(tx)=tJ(x)$ for all $t>0$ and $x\in X$, and $J(-x)=-J(x)$.

Let $U:=\{x\in X: \Vert x \Vert =1\}$. A Banach space *X* is said to be uniformly convex if for each $\varepsilon\in(0,2]$, there exists $\delta>0$ such that for any $x,y\in U$, $\Vert \frac{x+y}{2} \Vert >1-\delta\Rightarrow \Vert x-y \Vert <\varepsilon$. It is known that a uniformly convex Banach space is reflexive and strictly convex. Also, it is known that if a Banach space *X* is reflexive, then *X* is strictly convex if and only if $X^{*}$ is smooth as well as *X* is smooth if and only if $X^{*}$ is strictly convex.

### Proposition 1

[22]

*Let* *X* *be a smooth and uniformly convex Banach space*, *and let* $r>0$. *Then there exists a strictly increasing*, *continuous and convex function* $g:[0,2r]\to \mathbf{R}$, $g(0)=0$ *such that*

$$g\bigl( \Vert x-y \Vert \bigr)\leq \Vert x \Vert ^{2}-2\bigl\langle x,j(y)\bigr\rangle + \Vert y \Vert ^{2},\quad\forall x,y\in B_{r}, $$

*where* $B_{r}=\{x\in X: \Vert x \Vert \leq r\}$.

Here we define a function $\rho:[0,\infty)\to[0,\infty)$ called the modulus of smoothness of *X* as follows:

$$\rho(\tau)=\sup\biggl\{ \frac{1}{2}\bigl( \Vert x+y \Vert + \Vert x-y \Vert \bigr)-1:x,y\in X, \Vert x \Vert =1, \Vert y \Vert =\tau\biggr\} . $$

It is known that *X* is uniformly smooth if and only if $\lim_{\tau\to 0^{+}}\rho(\tau)/\tau=0$. Let *q* be a fixed real number with $1< q\leq2$. Then a Banach space *X* is said to be *q*-uniformly smooth if there exists a constant $c>0$ such that $\rho(\tau)\leq c\tau^{q}$ for all $\tau >0$. For further details on the geometry of Banach spaces, we refer to [1, 23] and the references therein. Takahashi et al. [24] reminded us of the fact that no Banach space is *q*-uniformly smooth for $q>2$. So, in this paper, we focus on only a 2-uniformly smooth Banach space as in [2].

### Lemma 1

[25]

*Let* *q* *be a given real number with* $1< q\leq2$, *and let* *X* *be a* *q*-*uniformly smooth Banach space*. *Then*

$$\Vert x+y \Vert ^{q}\leq \Vert x \Vert ^{q}+q\bigl\langle y,J_{q}(x)\bigr\rangle +2 \Vert \kappa y \Vert ^{q},\quad \forall x,y\in X, $$

*where* *κ* *is the* *q*-*uniformly smooth constant of* *X* *and* $J_{q}$ *is the generalized duality mapping from* *X* *into* $2^{X^{*}}$ *defined by*

$$J_{q}(x)=\bigl\{ \varphi\in X^{*}:\langle x,\varphi\rangle= \Vert x \Vert ^{q}, \Vert \varphi \Vert = \Vert x \Vert ^{q-1}\bigr\} ,\quad\forall x\in X. $$

Let *D* be a subset of *C*, and let Π be a mapping of *C* into *D*. Then Π is said to be sunny if

$$\Pi\bigl[\Pi(x)+t\bigl(x-\Pi(x)\bigr)\bigr]=\Pi(x), $$

whenever $\Pi(x)+t(x-\Pi(x))\in C$ for $x\in C$ and $t\geq0$. A mapping Π of *C* into itself is called a retraction if $\Pi^{2}=\Pi $. If a mapping Π of *C* into itself is a retraction, then $\Pi (z)=z$ for each $z\in R(\Pi)$, where $R(\Pi)$ is the range of Π. A subset *D* of *C* is called a sunny nonexpansive retract of *C* if there exists a sunny nonexpansive retraction from *C* onto *D*.

### Lemma 2

[26]

*Let* *C* *be a nonempty closed convex subset of a smooth Banach space* *X*, *D* *be a nonempty subset of* *C* *and* Π *be a retraction of* *C* *onto* *D*. *Then the following are equivalent*:

(i) Π *is sunny and nonexpansive*;
(ii) $\Vert \Pi(x)-\Pi(y) \Vert ^{2}\leq\langle x-y$, $j(\Pi(x)-\Pi (y))\rangle$, $\forall x,y\in C$;
(iii) $\langle x-\Pi(x),j(y-\Pi(x))\rangle\leq0$, $\forall x\in C$, $y\in D$.

It is well known that if *X* is a Hilbert space, then a sunny nonexpansive retraction $\Pi_{C}$ coincides with the metric projection from *X* onto *C*. Let *C* be a nonempty closed convex subset of a uniformly convex and uniformly smooth Banach space *X*, and let *T* be a nonexpansive mapping of *C* into itself with the fixed point set $\operatorname{Fix}(T)\neq\emptyset$. Then the set $\operatorname{Fix}(T)$ is a sunny nonexpansive retract of *C*; see, e.g., [2].

### Lemma 3

[2]

*Let* *C* *be a nonempty closed convex subset of a real* 2-*uniformly smooth Banach space* *X*. *Let* $\Pi_{C}$ *be a sunny nonexpansive retraction from* *X* *onto* *C*. *Let the mappings* $A,B:C\to X$ *be* *α*-*inverse*-*strongly accretive and* *β*-*inverse*-*strongly accretive*, *respectively*. *For given* $x^{*},y^{*}\in C$, $(x^{*},y^{*})$ *is a solution of GSVI* (2) *if and only if* $x^{*}\in\operatorname{GSVI}(C,A,B)$, *where* $\operatorname {GSVI}(C,A,B)$ *is the set of fixed points of the mapping* $G:=\Pi _{C}(I-\lambda A) \Pi_{C}(I-\mu B)$ *and* $y^{*}=\Pi_{C}(x^{*}-\mu Bx^{*})$.

### Proposition 2

[27]

*Let* *C* *be a nonempty closed convex subset of a real* 2-*uniformly smooth Banach space* *X*. *Let the mappings* $A,B:C\to X$ *be* *α*-*inverse*-*strongly accretive and* *β*-*inverse*-*strongly accretive*, *respectively*. *Then*

$$\bigl\Vert (I-\lambda A)x-(I-\lambda A)y \bigr\Vert ^{2}\leq \Vert x-y \Vert ^{2}+2\lambda\bigl(\kappa ^{2}\lambda- \alpha\bigr) \Vert Ax-Ay \Vert ^{2} $$

*and*

$$\bigl\Vert (I-\mu B)x-(I-\mu B)y \bigr\Vert ^{2}\leq \Vert x-y \Vert ^{2}+2\mu\bigl(\kappa^{2}\mu-\beta\bigr) \Vert Bx-By \Vert ^{2}. $$

*In particular*, *if* $0\leq\lambda\leq\frac{\alpha}{\kappa^{2}}$ *and* $0\leq \mu\leq\frac{\beta}{\kappa^{2}}$, *then* $I-\lambda A$ *and* $I-\mu B$ *are nonexpansive*.

### Lemma 4

[2]

*Let* *C* *be a nonempty closed convex subset of a real* 2-*uniformly smooth Banach space* *X*. *Let* $\Pi_{C}$ *be a sunny nonexpansive retraction from* *X* *onto* *C*. *Let the mappings* $A,B:C\to X$ *be* *α*-*inverse*-*strongly accretive and* *β*-*inverse*-*strongly accretive*, *respectively*. *Let the mapping* $G:C\to C$ *be defined as* $G:=\Pi_{C}(I-\lambda A)\Pi_{C}(I-\mu B)$. *If* $0\leq\lambda\leq\frac{\alpha}{\kappa^{2}}$ *and* $0\leq\mu\leq\frac {\beta}{\kappa^{2}}$, *then* $G:C\to C$ *is nonexpansive*.

Let *C* be a nonempty closed convex subset of a uniformly convex and 2-uniformly smooth Banach space *X*. Let $\Pi_{C}$ be a sunny nonexpansive retraction from *X* onto *C*. Let the mappings $A,B:C\to X$ be *α*-inverse-strongly accretive and *β*-inverse-strongly accretive, respectively. Let $F:C\to X$ be *δ*-strongly accretive and *ζ*-strictly pseudocontractive with $\delta+\zeta>1$. Assume that $\lambda\in(0,\frac{\alpha}{\kappa ^{2}})$ and $\mu\in(0,\frac{\beta}{\kappa^{2}})$, where *κ* is the 2-uniformly smooth constant of *X* (see Lemma 2). Very recently, in order to solve GSVI (2), Ceng et al. [3] introduced an implicit algorithm of Mann’s type.

### Algorithm 1

[3]

*For each* $t\in(0,1)$, *choose a number* $\theta_{t}\in(0,1)$ *arbitrarily*. *The net* $\{x_{t}\}$ *is generated by the implicit method*

$$x_{t}=t\Pi_{C}(I-\lambda A)\Pi_{C}(I-\mu B)x_{t}+(1-t)\Pi_{C}(I-\theta_{t}F)\Pi _{C}(I-\lambda A)\Pi_{C}(I-\mu B)x_{t},\quad \forall t\in(0,1), $$

*where* $x_{t}$ *is a unique fixed point of the contraction*

$$W_{t}=t\Pi_{C}(I-\lambda A)\Pi_{C}(I-\mu B)+(1-t)\Pi_{C}(I-\theta_{t}F)\Pi _{C}(I-\lambda A)\Pi_{C}(I-\mu B). $$

It was proven in [3] that the net $\{x_{t}\}$ converges in norm, as $t\to0^{+}$, to the unique solution $x^{*}\in\operatorname{GSVI}(C,A,B)$ to the following VI:

5 $$ \bigl\langle F\bigl(x^{*}\bigr),j\bigl(x-x^{*}\bigr)\bigr\rangle \geq0, \quad\forall x\in\operatorname {GSVI}(C,A,B), $$

provided $\lim_{t\to0^{+}}\theta_{t}=0$. In the meantime, the authors [3] also proposed another explicit algorithm of Mann’s type.

### Algorithm 2

[3]

*For arbitrarily given* $x_{0}\in C$, *let the sequence* $\{x_{k}\}$ *be generated iteratively by*

$$\begin{aligned} x_{k+1}={}&\beta_{k}x_{k}+\gamma_{k} \Pi_{C}(I-\lambda A)\Pi_{C}(I-\mu B)x_{k}\\ &{}+(1-\beta_{k}-\gamma_{k})\Pi_{C}(I- \lambda_{k}F)\Pi_{C}(I-\lambda A)\Pi _{C}(I-\mu B)x_{k}, \end{aligned}$$

*where* $\{\lambda_{k}\},\{\beta_{k}\}$ *and* $\{\gamma_{k}\}$ *are three sequences in* $[0,1]$ *such that* $\beta_{k}+\gamma_{k}\leq1$, $\forall k\geq0$.

On the other hand, a mapping *F* with domain $D(F)$ and range $R(F)$ in *X* is called

- accretive if for each $x,y\in D(F)$, there exists $j(x-y)\in J(x-y)$ such that
  $$\bigl\langle Fx-Fy,j(x-y)\bigr\rangle \geq0, $$
  where *J* is the normalized duality mapping;
- *δ*-strongly accretive if for each $x,y\in D(F)$, there exists $j(x-y)\in J(x-y)$ such that
  $$\bigl\langle Fx-Fy,j(x-y)\bigr\rangle \geq\delta \Vert x-y \Vert ^{2}\quad\mbox{for some }\delta\in(0,1); $$
- *α*-inverse-strongly accretive if for each $x,y\in D(F)$, there exists $j(x-y)\in J(x-y)$ such that
  $$\bigl\langle Fx-Fy,j(x-y)\bigr\rangle \geq\alpha \Vert Fx-Fy \Vert ^{2}\quad\mbox{for some }\alpha\in(0,1); $$
- *ζ*-strictly pseudocontractive if for each $x,y\in D(F)$, there exists $j(x-y)\in J(x-y)$ such that
  $$\bigl\langle Fx-Fy,j(x-y)\bigr\rangle \leq \Vert x-y \Vert ^{2}- \zeta \bigl\Vert x-y-(Fx-Fy) \bigr\Vert ^{2}\quad \mbox{for some } \zeta\in(0,1). $$

It is easy to see that (5) can be rewritten as (see [28])

6 $$ \bigl\langle (I-F)x-(I-F)y,j(x-y)\bigr\rangle \geq\zeta \bigl\Vert (I-F)x-(I-F)y \bigr\Vert ^{2}, $$

where *I* denotes the identity mapping of *X*. Clearly, if *F* is *ζ*-strictly pseudocontractive with $\zeta=0$, then it is said to be pseudocontractive. It is not hard to find that every nonexpansive mapping is pseudocontractive.

Let *C* be a nonempty closed convex subset of a smooth Banach space *X* and $\{T_{i}\}^{\infty}_{i=1}$ be an infinite family of nonexpansive self-mappings on *C*. Then we set ${\mathcal{F}}:=\bigcap^{\infty}_{i=1}\operatorname{Fix}(T_{i})$. In 2013, Buong and Phuong [18] considered the following HVI with $C=X$: find $x^{*}\in{\mathcal{F}}$ such that

7 $$ \bigl\langle F\bigl(x^{*}\bigr),j\bigl(x-x^{*}\bigr)\bigr\rangle \geq0, \quad\forall x\in{\mathcal{F}}. $$

In the case where $X=H$, a Hilbert space, we have $J=I$, and hence problem (7) reduces to the HVI: find $x^{*}\in{\mathcal{F}}$ such that

8 $$ \bigl\langle F\bigl(x^{*}\bigr),x-x^{*}\bigr\rangle \geq0,\quad\forall x\in{\mathcal{F}}. $$

Assume that ${\mathcal{F}}=\bigcap^{N}_{i=1}\operatorname{Fix}(T_{i})$ is the set of common fixed points of a family of *N* nonexpansive mappings $T_{i}$ on *H*, and *F* is an *L*-Lipschitz continuous and *η*-strongly monotone mapping, i.e., $\Vert Fx-Fy \Vert \leq L \Vert x-y \Vert $ and $\langle Fx-Fy,x-y\rangle\geq\eta \Vert x-y \Vert ^{2}$ for all $x,y\in H$. Zeng and Yao [19] introduced the following implicit iteration: for an arbitrarily initial point $x_{0}\in H$, the sequence $\{x_{k}\}^{\infty}_{k=1}$ is generated as follows:

9 $$ x_{k}=\beta_{k}x_{k-1}+(1- \beta_{k})\bigl[T_{[k]}x_{k}-\lambda_{k}\mu F(T_{[k]}x_{k})\bigr],\quad\forall k\geq1, $$

where $T_{[n]}=T_{n\operatorname{mod}N}$, for integer $n\geq1$, with the mod function taking values in the set $\{1,2,\ldots,N\}$. They proved the following result.

### Theorem 3

[19]

*Let* *H* *be a real Hilbert space*, *and let* $F:H\to H$ *be a mapping such that*, *for some positive constants* *L* *and* *η*, *F* *is* *L*-*Lipschitz continuous and* *η*-*strongly monotone*. *Let* $\{T_{i}\}^{N}_{i=1}$ *be* *N* *nonexpansive mappings on* *H* *such that* ${\mathcal{F}}:\bigcap^{N}_{i=1}\operatorname{Fix}(T_{i})\neq\emptyset$. *Let* $\mu\in(0,2\eta /L^{2})$, $x_{0}\in H$, $\{\lambda_{k}\}^{\infty}_{k=1}\subset[0,1)$ *and* $\{\beta_{k}\}^{\infty}_{k=1}\subset(0,1)$ *satisfying the conditions* $\sum^{\infty}_{k=1}\lambda_{k}<\infty$, *and let* $a\leq\beta_{k}\leq b,k\geq1$, *for some* $a,b\in(0,1)$. *Then the sequence* $\{x_{k}\}^{\infty}_{k=0}$, *defined by* (9), *converges weakly to* $x^{*}\in{\mathcal{F}}$.

Recently, for deriving the strong convergence and weakening the condition on $\lambda_{k}$, Buong and Anh [21] proposed the following implicit iteration method:

10 $$ x_{t}=T^{t}x_{t},\quad T^{t}:=T^{t}_{0}T^{t}_{N} \cdots T^{t}_{1}, t\in(0,1), $$

where the sequence $\{T^{t}_{i}\}^{N}_{i=0}$ is defined by

11 $$ \textstyle\begin{cases} T^{t}_{i}x:=(1-\beta^{i}_{t})x+\beta^{i}_{t}T_{i}x,& i=1,\ldots,N,\\ T^{t}_{0}y:=(I-\lambda_{t}\mu F)y,& x,y\in H, \end{cases} $$

and proved that the nets $\{x_{t}\}$, defined by (10)-(11), converge strongly to an element $x^{*}$ in (8). Under the assumptions that $N=1$, *X* is a real reflexive and strictly convex Banach space with a uniformly Gâteaux differentiable norm, and *T* is a continuous pseudocontractive mapping, Ceng et al. [29] proved the following result.

### Theorem 4

[29]

*Let* *F* *be a* *δ*-*strongly accretive and* *ζ*-*strictly pseudocontractive mapping with* $\delta+\zeta>1$, *and let* *T* *be a continuous and pseudocontractive mapping on* *X*, *which is a real reflexive and strictly convex Banach space with a uniformly Gâteaux differentiable norm such that* ${\mathcal{F}}:=\operatorname{Fix}(T)\neq \emptyset$. *For each* $t\in(0,1)$, *choose a number* $\mu_{t}\in(0,1)$ *arbitrarily*, *and let* $\{z_{t}\}$ *be defined by*

12 $$ z_{t}=t(I-\mu_{t}F)z_{t}+(1-t)Tz_{t}. $$

*Then*, *as* $t\to0^{+}$, $\{z_{t}\}$ *converges strongly to* $x^{*}\in{\mathcal{F}}$.

In [30], Takahashi introduced the following *W*-mapping which is generated by $T_{k},T_{k-1}, \ldots,T_{1}$ and real numbers $\alpha_{k},\alpha _{k-1},\ldots,\alpha_{1}$ as follows:

13 $$ \textstyle\begin{cases} U_{k,k+1}=I,\\ U_{k,k}=\alpha_{k}T_{k}U_{k,k+1}+(1-\alpha_{k})I,\\ U_{k,k-1}=\alpha_{k-1}T_{k-1}U_{k,k}+(1-\alpha_{k-1})I,\\ \cdots\\ U_{k,2}=\alpha_{2}T_{2}U_{k,3}+(1-\alpha_{2})I,\\ W_{k}=U_{k,1}=\alpha_{1}T_{1}U_{k,2}+(1-\alpha_{1})I. \end{cases} $$

Kikkawa and Takahashi [31] considered the following strongly convergent implicit method:

14 $$ S_{k}x=\biggl(1-\frac{1}{k}\biggr)Ux+ \frac{1}{k}f(x),\quad\mbox{and}\quad Ux=\lim_{k\to\infty}W_{k}x= \lim_{k\to\infty}U_{k,1}x. $$

However, the method (14) is very difficult to be grasped due to the limit mapping *U*.

Motivated by methods (10) and (12), Buong and Phuong [18] considered two implicit methods by introducing a mapping $V_{k}$ defined by

15 $$ V_{k}=V^{1}_{k},\qquad V^{i}_{k}=T^{i}T^{i+1}\cdots T^{k},\qquad T^{i}=(1-\alpha _{i})I+ \alpha_{i}T_{i},\quad i=1,2,\ldots,k, $$

where

16 $$ \alpha_{i}\in(0,1)\quad\mbox{and}\quad\sum ^{\infty}_{i=1}\alpha_{i}< \infty. $$

In both methods, the iteration sequence $\{x_{k}\}^{\infty}_{k=1}$ is defined, respectively, by

17 $$ x_{k}=V_{k}(I-\lambda_{k}F)x_{k}, \quad\forall k\geq1, $$

and

18 $$ x_{k}=\gamma_{k}(I-\lambda_{k}F)x_{k}+(I- \gamma_{k})V_{k}x_{k},\quad\forall k\geq1, $$

where $\lambda_{k}$ and $\gamma_{k}$ are the positive parameters satisfying some additional conditions. The strong convergence theorems for methods (17) and (18) are also established.

We will make use of the following well-known results.

### Lemma 5

*Let* *X* *be a real normed linear space*. *Then the following inequality holds*:

$$\Vert x+y \Vert ^{2}\leq \Vert x \Vert ^{2}+2\bigl\langle y,j(x+y)\bigr\rangle ,\quad\forall x,y\in X,\forall j(x+y)\in J(x+y). $$

### Lemma 6

[32]

*Let* *C* *be a nonempty closed convex subset of a uniformly convex Banach space* *X* *and* $T:C\to C$ *be a nonexpansive mapping with* $\operatorname{Fix}(T)\neq\emptyset$. *If* $\{ x_{n}\}$ *is a sequence of* *C* *such that* $x_{n}\rightharpoonup x$ *and* $(I-T)x_{n}\to y$, *then* $(I-T)x=y$. *In particular*, *if* $y=0$, *then* $x\in \operatorname{Fix}(T)$.

### Lemma 7

[2]

*Let* *C* *be a nonempty closed convex subset of a real smooth Banach space* *X*. *Assume that the mapping* $F:C\to X$ *is accretive and weakly continuous along segments* (*that is*, $F(x+ty)\rightharpoonup F(x)$ *as* $t\to0$). *Then the variational inequality*

$$x^{*}\in C,\quad\bigl\langle F\bigl(x^{*}\bigr),j\bigl(x-x^{*}\bigr)\bigr\rangle \geq0, \quad\forall x\in C $$

*is equivalent to the following Minty type variational inequality*:

$$x^{*}\in C,\quad\bigl\langle F(x),j\bigl(x-x^{*}\bigr)\bigr\rangle \geq0,\quad\forall x\in C. $$

### Lemma 8

[3]

*Let* *X* *be a real smooth Banach space and* $F:C\to X$ *be a mapping*.

- *If* *F* *is* *ζ*-*strictly pseudocontractive*, *then* *F* *is Lipschitz continuous with constant* $1+\frac{1}{\zeta}$.
- *If* *F* *is* *δ*-*strongly accretive and* *ζ*-*strictly pseudocontractive with* $\delta+\zeta>1$, *then* $I-F$ *is contractive with constant* $\sqrt{\frac{1-\delta}{\zeta}}\in(0,1)$.
- *If* *F* *is* *δ*-*strongly accretive and* *ζ*-*strictly pseudocontractive with* $\delta+\zeta>1$, *then for any fixed number* $\lambda\in(0,1)$, $I-\lambda F$ *is contractive with constant* $1-\lambda(1-\sqrt{\frac{1-\delta}{\zeta}})\in(0,1)$.

## Iterative algorithms and convergence criteria

In this section, we study iterative methods for computing approximate solutions of the HVI (for an infinite family of nonexpansive mappings) with a GSVI constraint. We introduce implicit and explicit iterative algorithms for solving such a problem. We show the strong convergence theorems for the sequences generated by the proposed algorithms.

The following lemmas will be used to prove our main results in the sequel.

### Lemma 9

[18]

*Let* *C* *be a nonempty closed convex subset of a strictly convex Banach space* *X*, *and let* $\{T_{i}\}^{k}_{i=1},k\geq1$, *be* *k* *nonexpansive self*-*mappings on* *C* *such that the set of common fixed points* ${\mathcal{F}}:=\bigcap^{k}_{i=1}\operatorname{Fix}(T_{i})\neq\emptyset$. *Let* $a,b$ *and* $\alpha _{i},i=1,2,\ldots,k$, *be real numbers such that* $0< a\leq\alpha_{i}\leq b<1$, *and let* $V_{k}$ *be a mapping defined by* (15) *for all* $k\geq1$. *Then* $\operatorname{Fix}(V_{k})={\mathcal{F}}$.

### Lemma 10

[18]

*Let* *C* *be a nonempty closed convex subset of a Banach space* *X*, *and let* $\{T_{i}\}^{\infty}_{i=1}$ *be an infinite family of nonexpansive self*-*mappings on* *C* *such that the set of common fixed points* ${\mathcal{F}}:=\bigcap^{\infty}_{i=1}\operatorname {Fix}(T_{i})\neq\emptyset$. *Let* $V_{k}$ *be a mapping defined by* (15), *and let* $\alpha_{i}$ *satisfy* (16). *Then*, *for each* $x\in C$ *and* $i\geq1$, $\lim_{k\to\infty }V^{i}_{k}x$ *exists*.

### Remark 1

(i) We can define the mappings
  $$V^{i}_{\infty}x:=\lim_{k\to\infty}V^{i}_{k}x \quad\mbox{and}\quad Vx:=V^{1}_{\infty}x=\lim _{k\to\infty}V_{k}x,\quad\forall x\in C. $$
(ii) It can be readily seen from the proof of Lemma 10 that if *D* is a nonempty and bounded subset of *C*, then the following holds:
  $$\lim_{k\to\infty}\sup_{x\in D} \bigl\Vert V^{i}_{k}x-V^{i}_{\infty}x \bigr\Vert =0, \quad\forall i\geq1. $$
  In particular, whenever $i=1$, we have
  $$\lim_{k\to\infty}\sup_{x\in D} \Vert V_{k}x-Vx \Vert =0. $$

### Lemma 11

[18]

*Let* *C* *be a nonempty closed convex subset of a strictly convex Banach space* *X*, *and let* $\{T_{i}\}^{\infty}_{i=1}$ *be an infinite family of nonexpansive self*-*mappings on* *C* *such that the set of common fixed points* ${\mathcal{F}}:=\bigcap^{\infty}_{i=1}\operatorname{Fix}(T_{i})\neq\emptyset $. *Let* $\alpha_{i}$ *satisfy the first condition in* (16). *Then* $\operatorname{Fix}(V)={\mathcal{F}}$.

### Lemma 12

[33]

*Let* $\{x_{n}\}$ *and* $\{z_{n}\}$ *be bounded sequences in a Banach space* *X*, *and let* $\{\alpha_{k}\}$ *be a sequence in* $[0,1]$ *such that*

$$0< \liminf_{k\to\infty}\alpha_{k}\leq\limsup _{k\to\infty}\alpha_{k}< 1. $$

*Suppose that* $x_{k+1}=\alpha_{k}x_{k}+(1-\alpha_{k})z_{k},\forall k\geq1$, *and*

$$\limsup_{k\to\infty}\bigl( \Vert z_{k+1}-z_{k} \Vert - \Vert x_{k+1}-x_{k} \Vert \bigr)\leq0. $$

*Then* $\lim_{k\to\infty} \Vert z_{k}-x_{k} \Vert =0$.

### Lemma 13

[34]

*Assume that* $\{a_{k}\}$ *is a sequence of nonnegative real numbers such that*

$$a_{k+1}\leq(1-\gamma_{k})a_{k}+\gamma_{k} \delta_{k},\quad\forall k\geq1, $$

*where* $\{\gamma_{k}\}$ *is a sequence in* $[0,1]$ *and* $\{\delta_{k}\}$ *is a sequence in* **R** *such that*

(i) $\sum^{\infty}_{k=1}\gamma_{k}=\infty$;
(ii) $\limsup_{k\to\infty}\delta_{k}\leq0$ *or* $\sum^{\infty}_{k=1}|\gamma_{k}\delta_{k}|<\infty$.

*Then* $\lim_{k\to\infty}a_{k}=0$.

Now, we are in a position to prove the following main results.

### Theorem 5

*Let* *C* *be a nonempty closed convex subset of a uniformly convex and* 2-*uniformly smooth Banach space* *X*. *Let* $\Pi_{C}$ *be a sunny nonexpansive retraction from* *X* *onto* *C*. *Let the mappings* $A,B:C\to X$ *be* *α*-*inverse*-*strongly accretive and* *β*-*inverse*-*strongly accretive*, *respectively*. *Let* $F:C\to X$ *be* *δ*-*strongly accretive and* *ζ*-*strictly pseudocontractive with* $\delta+\zeta>1$. *Assume that* $\lambda\in(0,\frac {\alpha}{\kappa^{2}})$ *and* $\mu\in(0,\frac{\beta}{\kappa^{2}})$ *where* *κ* *is the* 2-*uniformly smooth constant of* *X*. *Let* $\{T_{i}\} ^{\infty}_{i=1}$ *be an infinite family of nonexpansive self*-*mappings on* *C* *such that* ${\mathcal{F}}:=\bigcap^{\infty}_{i=1}\operatorname{Fix}(T_{i})\cap\operatorname{GSVI}(C,A,B)\neq\emptyset $. *Let* $\{V_{k}\} ^{\infty}_{k=1}$ *be defined by* (15) *and* (16). *Let* $\{x_{k}\} ^{\infty}_{k=1}$ *be defined by*

$$\begin{aligned} x_{k} =& \gamma_{k}\Pi_{C}(I- \lambda_{k}F)\Pi_{C}(I-\lambda A)\Pi_{C}(I-\mu B)V_{k}x_{k} \\ &{} +(1-\gamma_{k})\Pi_{C}(I-\lambda A) \Pi_{C}(I-\mu B)V_{k}x_{k},\quad \forall k\geq1, \end{aligned}$$

*where* $\{\gamma_{k}\}$ *and* $\{\lambda_{k}\}$ *are sequences in* $(0,1]$ *such that* $\gamma_{k}\to0$ *and* $\lambda_{k}\to0$ *as* $k\to\infty$. *Then* $\{x_{k}\}^{\infty}_{k=1}$ *converges strongly to a unique solution* $x^{*}\in {\mathcal{F}}$ *to the following VI*:

19 $$ \bigl\langle F\bigl(x^{*}\bigr),j\bigl(x-x^{*}\bigr)\bigr\rangle \geq0, \quad\forall x\in{\mathcal{F}}. $$

### Proof

Let the mapping $G:C\to C$ be defined as $G:=\Pi_{C}(I-\lambda A)\Pi _{C}(I-\mu B)$, where $0<\lambda<\frac{\alpha}{\kappa^{2}}$ and $0<\mu<\frac {\beta}{\kappa^{2}}$. In terms of Lemma 4 we know that $G:C\to C$ is nonexpansive. Then the implicit iterative scheme can be rewritten as

20 $$ x_{k}=\gamma_{k}\Pi_{C}(I- \lambda_{k}F)GV_{k}x_{k}+(1-\gamma_{k})GV_{k}x_{k}, \quad \forall k\geq1. $$

Consider the mapping $U_{k}x=\gamma_{k}\Pi_{C}(I-\lambda_{k}F)GV_{k}x+(1-\gamma _{k})GV_{k}x,\forall x\in C$. From Lemmas 4 and 8(c), it follows that for each $x,y\in C$,

$$\begin{aligned} \Vert U_{k}x-U_{k}y \Vert =& \bigl\Vert \gamma_{k}\bigl(\Pi_{C}(I-\lambda_{k}F)GV_{k}x- \Pi_{C}(I-\lambda_{k}F)GV_{k}y\bigr)\\ &{}+(1- \gamma_{k}) (GV_{k}x-GV_{k}y) \bigr\Vert \\ \leq& \gamma_{k} \bigl\Vert (I-\lambda_{k}F)GV_{k}x-(I- \lambda_{k}F)GV_{k}y \bigr\Vert +(1-\gamma _{k}) \Vert GV_{k}x-GV_{k}y \Vert \\ \leq& \gamma_{k}(1-\lambda_{k}\tau) \Vert GV_{k}x-GV_{k}y \Vert +(1-\gamma_{k}) \Vert GV_{k}x-GV_{k}y \Vert \\ =& (1-\gamma_{k}\lambda_{k}\tau) \Vert GV_{k}x-GV_{k}y \Vert \\ \leq& (1-\gamma_{k}\lambda_{k}\tau) \Vert x-y \Vert , \end{aligned}$$

where $\tau=1-\sqrt{\frac{1-\delta}{\zeta}}\in(0,1)$ (by using $\delta +\zeta>1$). Due to $\gamma_{k}\lambda_{k}\tau\in(0,1)$, $U_{k}$ is a contraction of *C* into itself. Hence, by Banach’s contraction principle, there exists a unique element $x_{k}\in C$ satisfying (20).

Next, we divide the rest of the proof into several steps.

*Step 1*. We show that $\{x_{k}\}^{\infty}_{k=1}$ is bounded. Indeed, take an arbitrarily given $p\in{\mathcal{F}}$. Then we have $V_{k}p=p$ and $Gp=p$. Hence, by Lemma 8(c) we get

21 $$\begin{aligned} \Vert x_{k}-p \Vert ^{2} =& \bigl\Vert \gamma_{k}\Pi_{C}(I-\lambda_{k}F)GV_{k}x_{k}+(1- \gamma_{k})GV_{k}x_{k}-p \bigr\Vert ^{2} \\ \leq& \gamma_{k} \bigl\Vert \Pi_{C}(I- \lambda_{k}F)GV_{k}x_{k}-p \bigr\Vert ^{2}+(1-\gamma_{k}) \Vert GV_{k}x_{k}-p \Vert ^{2} \\ \leq& \gamma_{k} \bigl\Vert (I-\lambda_{k}F)GV_{k}x_{k}-p \bigr\Vert ^{2}+(1-\gamma_{k}) \Vert GV_{k}x_{k}-p \Vert ^{2} \\ =& \gamma_{k} \bigl\Vert (I-\lambda_{k}F)GV_{k}x_{k}-(I- \lambda_{k}F)p-\lambda_{k}F(p) \bigr\Vert ^{2}+(1- \gamma_{k}) \Vert GV_{k}x_{k}-p \Vert ^{2} \\ \leq& \gamma_{k}\bigl[(1-\lambda_{k}\tau) \Vert GV_{k}x_{k}-p \Vert +\lambda_{k} \bigl\Vert F(p) \bigr\Vert \bigr]^{2}+(1-\gamma_{k}) \Vert GV_{k}x_{k}-p \Vert ^{2} \\ \leq& \gamma_{k}\bigl[(1-\lambda_{k}\tau) \Vert GV_{k}x_{k}-p \Vert ^{2}+\lambda_{k} \tau^{-1} \bigl\Vert F(p) \bigr\Vert ^{2}\bigr]+(1- \gamma_{k}) \Vert GV_{k}x_{k}-p \Vert ^{2} \\ =& (1-\gamma_{k}\lambda_{k}\tau) \Vert GV_{k}x_{k}-p \Vert ^{2}+\gamma_{k} \lambda_{k}\tau ^{-1} \bigl\Vert F(p) \bigr\Vert ^{2} \\ \leq& (1-\gamma_{k}\lambda_{k}\tau) \Vert x_{k}-p \Vert ^{2}+\gamma_{k} \lambda_{k}\tau ^{-1} \bigl\Vert F(p) \bigr\Vert ^{2}. \end{aligned}$$

Therefore, $\Vert x_{k}-p \Vert \leq \Vert F(p) \Vert /\tau$, which also leads to the boundedness of $\{x_{k}\}^{\infty}_{k=1}$. So, the sequences $\{V_{k}x_{k}\} ^{\infty}_{k=1}$, $\{y_{k}\}^{\infty}_{k=1}$ and $\{F(y_{k})\}^{\infty}_{k=1}$, where $y_{k}=GV_{k}x_{k}$, are also bounded. Since $\gamma_{k}\to0$ as $k\to\infty$, and the following relation holds

$$\begin{aligned} \Vert x_{k}-GV_{k}x_{k} \Vert =& \gamma_{k} \bigl\Vert \Pi_{C}(I-\lambda_{k}F)GV_{k}x_{k}-GV_{k}x_{k} \bigr\Vert \\ \leq& \gamma_{k} \bigl\Vert (I-\lambda_{k}F)GV_{k}x_{k}-GV_{k}x_{k} \bigr\Vert \\ =& \gamma_{k}\lambda_{k} \bigl\Vert F(y_{k}) \bigr\Vert \leq\gamma_{k} \bigl\Vert F(y_{k}) \bigr\Vert , \end{aligned}$$

we obtain from the boundedness of $\{F(y_{k})\}$ that $\Vert x_{k}-GV_{k}x_{k} \Vert \to 0$ as $k\to\infty$.

*Step 2.* We show that $\Vert z_{k}-Gz_{k} \Vert \to0$ as $k\to\infty$, where $z_{k}=V_{k}x_{k}$ for all $k\geq1$. Indeed, for simplicity, put $q=\Pi_{C}(p-\mu Bp)$ and $u_{k}=\Pi_{C}(z_{k}-\mu Bz_{k})$. Then $y_{k}=Gz_{k}=\Pi_{C}(u_{k}- \lambda Au_{k})$ for all $k\geq1$. From Lemma 2, we have

22 $$\begin{aligned} \Vert u_{k}-q \Vert ^{2} =& \bigl\Vert \Pi_{C}(z_{k}-\mu Bz_{k})- \Pi_{C}(p-\mu Bp) \bigr\Vert ^{2} \\ \leq& \bigl\Vert z_{k}-p-\mu(Bz_{k}-Bp) \bigr\Vert ^{2} \\ \leq& \Vert z_{k}-p \Vert ^{2}-2\mu\bigl(\beta- \kappa^{2}\mu\bigr) \Vert Bz_{k}-Bp \Vert ^{2} \\ \leq& \Vert x_{k}-p \Vert ^{2}-2\mu\bigl(\beta- \kappa^{2}\mu\bigr) \Vert Bz_{k}-Bp \Vert ^{2}, \end{aligned}$$

and

23 $$\begin{aligned} \Vert y_{k}-p \Vert ^{2} =& \bigl\Vert \Pi_{C}(u_{k}-\lambda Au_{k})- \Pi_{C}(q-\lambda Aq) \bigr\Vert ^{2} \\ \leq& \bigl\Vert u_{k}-q-\lambda(Au_{k}-Aq) \bigr\Vert ^{2} \\ \leq& \Vert u_{k}-q \Vert ^{2}-2\lambda\bigl(\alpha- \kappa^{2}\lambda\bigr) \Vert Au_{k}-Aq \Vert ^{2}. \end{aligned}$$

Substituting (22) for (23), we obtain

24 $$\begin{aligned} \Vert y_{k}-p \Vert ^{2}\leq {}&\Vert x_{k}-p \Vert ^{2}-2\mu\bigl(\beta-\kappa^{2}\mu \bigr) \Vert Bz_{k}-Bp \Vert ^{2} \\ &{}-2\lambda \bigl(\alpha- \kappa^{2}\lambda\bigr) \Vert Au_{k}-Aq \Vert ^{2}. \end{aligned}$$

From (21) and (24), we have

$$\begin{aligned} \Vert x_{k}-p \Vert ^{2} \leq& (1- \gamma_{k}\lambda_{k}\tau) \Vert Gz_{k}-p \Vert ^{2}+\gamma _{k}\lambda_{k}\tau^{-1} \bigl\Vert F(p) \bigr\Vert ^{2} \\ \leq& \Vert Gz_{k}-p \Vert ^{2}+\gamma_{k} \tau^{-1} \bigl\Vert F(p) \bigr\Vert ^{2} \\ \leq& \Vert x_{k}-p \Vert ^{2}-2\mu\bigl(\beta- \kappa^{2}\mu\bigr) \Vert Bz_{k}-Bp \Vert ^{2} \\ &{} -2\lambda\bigl(\alpha-\kappa^{2}\lambda\bigr) \Vert Au_{k}-Aq \Vert ^{2}+\gamma_{k} \frac{ \Vert F(p) \Vert ^{2}}{\tau}, \end{aligned}$$

which immediately yields

$$2\mu\bigl(\beta-\kappa^{2}\mu\bigr) \Vert Bz_{k}-Bp \Vert ^{2}+2\lambda\bigl(\alpha-\kappa^{2}\lambda\bigr) \Vert Au_{k}-Aq \Vert ^{2}\leq\gamma_{k} \frac{ \Vert F(p) \Vert ^{2}}{\tau}. $$

So, from $\lambda\in(0,\frac{\alpha}{\kappa^{2}})$, $\mu\in(0,\frac {\beta}{\kappa^{2}})$ and $\gamma_{k}\to0$ as $k\to\infty$, we deduce that

25 $$ \lim_{k\to\infty} \Vert Bz_{k}-Bp \Vert =0\quad\mbox{and}\quad\lim_{k\to\infty} \Vert Au_{k}-Aq \Vert =0. $$

Utilizing Proposition 1 and Lemma 2, we have

$$\begin{aligned} \Vert u_{k}-q \Vert ^{2} =& \bigl\Vert \Pi_{C}(z_{k}-\mu Bz_{k})-\Pi_{C}(p-\mu Bp) \bigr\Vert ^{2} \\ \leq& \bigl\langle (z_{k}-\mu Bz_{k})-(p-\mu Bp),j(u_{k}-q)\bigr\rangle \\ =& \bigl\langle (z_{k}-p,j(u_{k}-q)\bigr\rangle +\mu\bigl\langle Bp-Bz_{k},j(u_{k}-q)\bigr\rangle \\ \leq& \frac{1}{2}\bigl[ \Vert z_{k}-p \Vert ^{2}+ \Vert u_{k}-q \Vert ^{2}-g_{1}\bigl( \bigl\Vert z_{k}-u_{k}-(p-q) \bigr\Vert \bigr)\bigr]\\ &{}+\mu \Vert Bp-Bz_{k} \Vert \Vert u_{k}-q \Vert \\ \leq& \frac{1}{2}\bigl[ \Vert x_{k}-p \Vert ^{2}+ \Vert u_{k}-q \Vert ^{2}-g_{1}\bigl( \bigl\Vert z_{k}-u_{k}-(p-q) \bigr\Vert \bigr)\bigr]\\ &{}+\mu \Vert Bp-Bz_{k} \Vert \Vert u_{k}-q \Vert , \end{aligned}$$

which implies that

26 $$\begin{aligned} \Vert u_{k}-q \Vert ^{2}\leq{}& \Vert x_{k}-p \Vert ^{2}-g_{1}\bigl( \bigl\Vert z_{k}-u_{k}-(p-q) \bigr\Vert \bigr) \\ &{}+2\mu \Vert Bp-Bz_{k} \Vert \Vert u_{k}-q \Vert . \end{aligned}$$

In the same way, we derive

$$\begin{aligned} \Vert y_{k}-p \Vert ^{2} =& \bigl\Vert \Pi_{C}(u_{k}-\lambda Au_{k})-\Pi_{C}(q- \lambda Aq) \bigr\Vert ^{2} \\ \leq& \bigl\langle u_{k}-\lambda Au_{k}-(q-\lambda Aq),j(y_{k}-p)\bigr\rangle \\ =& \bigl\langle u_{k}-q,j(y_{k}-p)\bigr\rangle +\lambda\bigl\langle Aq-Au_{k},j(y_{k}-p)\bigr\rangle \\ \leq& \frac{1}{2}\bigl[ \Vert u_{k}-q \Vert ^{2}+ \Vert y_{k}-p \Vert ^{2}-g_{2}\bigl( \bigl\Vert u_{k}-y_{k}+(p-q) \bigr\Vert \bigr)\bigr]\\ &{}+\lambda \Vert Aq-Au_{k} \Vert \Vert y_{k}-p \Vert , \end{aligned}$$

which implies that

27 $$ \Vert y_{k}-p \Vert ^{2}\leq \Vert u_{k}-q \Vert ^{2}-g_{2}\bigl( \bigl\Vert u_{k}-y_{k}+(p-q) \bigr\Vert \bigr)+2\lambda \Vert Aq-Au_{k} \Vert \Vert y_{k}-p \Vert . $$

Substituting (26) for (27), we get

28 $$\begin{aligned} \Vert y_{k}-p \Vert ^{2} \leq& \Vert x_{k}-p \Vert ^{2}-g_{1}\bigl( \bigl\Vert z_{k}-u_{k}-(p-q) \bigr\Vert \bigr)-g_{2} \bigl( \bigl\Vert u_{k}-y_{k}+(p-q) \bigr\Vert \bigr) \\ &{}+2\mu \Vert Bp-Bz_{k} \Vert \Vert u_{k}-q \Vert +2\lambda \Vert Aq-Au_{k} \Vert \Vert y_{k}-p \Vert . \end{aligned}$$

So, from (21) and (28) it follows that

$$\begin{aligned} \Vert x_{k}-p \Vert ^{2} \leq& (1- \gamma_{k}\lambda_{k}\tau) \Vert Gz_{k}-p \Vert ^{2}+\gamma _{k}\lambda_{k}\tau^{-1} \bigl\Vert F(p) \bigr\Vert ^{2} \\ \leq& \Vert y_{k}-p \Vert ^{2}+\gamma_{k} \frac{ \Vert F(p) \Vert ^{2}}{\tau} \\ \leq& \Vert x_{k}-p \Vert ^{2}-g_{1}\bigl( \bigl\Vert z_{k}-u_{k}-(p-q) \bigr\Vert \bigr)-g_{2}\bigl( \bigl\Vert u_{k}-y_{k}+(p-q) \bigr\Vert \bigr) \\ &{}+ 2\mu \Vert Bp-Bz_{k} \Vert \Vert u_{k}-q \Vert +2\lambda \Vert Aq-Au_{k} \Vert \Vert y_{k}-p \Vert +\gamma _{k}\frac{ \Vert F(p) \Vert ^{2}}{\tau}, \end{aligned}$$

which hence leads to

29 $$\begin{aligned} & g_{1}\bigl( \bigl\Vert z_{k}-u_{k}-(p-q) \bigr\Vert \bigr)+g_{2}\bigl( \bigl\Vert u_{k}-y_{k}+(p-q) \bigr\Vert \bigr) \\ &\quad\leq2\mu \Vert Bp-Bz_{k} \Vert \Vert u_{k}-q \Vert +2\lambda \Vert Aq-Au_{k} \Vert \Vert y_{k}-p \Vert +\gamma _{k}\frac{ \Vert F(p) \Vert ^{2}}{\tau}. \end{aligned}$$

From (25), $\gamma_{k}\to0$ as $k\to\infty$, and the boundedness of $\{u_{k}\}$ and $\{y_{k}\}$, we deduce that

$$\lim_{k\to\infty}g_{1}\bigl( \bigl\Vert z_{k}-u_{k}-(p-q) \bigr\Vert \bigr)=0\quad\mbox{and}\quad \lim_{k\to \infty}g_{2}\bigl( \bigl\Vert u_{k}-y_{k}+(p-q) \bigr\Vert \bigr)=0. $$

Utilizing the properties of $g_{1}$ and $g_{2}$, we conclude that

30 $$ \lim_{k\to\infty} \bigl\Vert z_{k}-u_{k}-(p-q) \bigr\Vert =0\quad\mbox{and}\quad\lim_{k\to\infty } \bigl\Vert u_{k}-y_{k}+(p-q) \bigr\Vert =0. $$

From (30), we get

$$\Vert z_{k}-y_{k} \Vert \leq \bigl\Vert z_{k}-u_{k}-(p-q) \bigr\Vert + \bigl\Vert u_{k}-y_{k}+(p-q) \bigr\Vert \to0\quad\mbox{as } k\to \infty. $$

That is,

31 $$ \lim_{k\to\infty} \Vert z_{k}-Gz_{k} \Vert =\lim_{k\to\infty} \Vert z_{k}-y_{k} \Vert =0. $$

This together with $\Vert x_{k}-GV_{k}x_{k} \Vert \to0$ implies that

32 $$ \lim_{k\to\infty} \Vert x_{k}-y_{k} \Vert =0\quad\mbox{and}\quad\lim_{k\to\infty} \Vert x_{k}-V_{k}x_{k} \Vert =\lim_{k\to\infty} \Vert x_{k}-z_{k} \Vert =0. $$

*Step 3.* We show that $\omega_{w}(x_{k})\subset{\mathcal{F}}$, where

$$\omega_{w}(x_{k})=\bigl\{ x\in C:x_{k_{i}} \rightharpoonup x \mbox{ for some subsequence }\{x_{k_{i}}\}\mbox{ of } \{x_{k}\}\bigr\} . $$

Indeed, we first claim that $\Vert x_{k}-Vx_{k} \Vert \to0$ as $k\to\infty$. It is easy to see from Remark 1(ii) that if *D* is a nonempty and bounded subset of *C*, then, for $\varepsilon>0$, there exists $k_{0}>i$ such that for all $k>k_{0}$

$$\sup_{x\in D} \bigl\Vert V^{i}_{k}x-V^{i}_{\infty}x \bigr\Vert \leq\varepsilon. $$

Taking $D=\{x_{k}:k\geq1\}$ and $i=1$, we have

$$\Vert V_{k}x_{k}-Vx_{k} \Vert \leq\sup _{x\in D} \Vert V_{k}x-Vx \Vert \leq\varepsilon. $$

So, it follows that

33 $$ \lim_{k\to\infty} \Vert V_{k}x_{k}-Vx_{k} \Vert =0. $$

Noting that

34 $$\begin{aligned} \Vert GVx_{k}-Vx_{k} \Vert \leq& \Vert GVx_{k}-GV_{k}x_{k} \Vert + \Vert GV_{k}x_{k}-V_{k}x_{k} \Vert + \Vert V_{k}x_{k}-Vx_{k} \Vert \\ \leq& \Vert Vx_{k}-V_{k}x_{k} \Vert + \Vert Gz_{k}-z_{k} \Vert + \Vert V_{k}x_{k}-Vx_{k} \Vert \\ =& 2 \Vert V_{k}x_{k}-Vx_{k} \Vert + \Vert Gz_{k}-z_{k} \Vert , \end{aligned}$$

from (31), (33) and (34) we obtain that

35 $$ \lim_{k\to\infty} \Vert GVx_{k}-Vx_{k} \Vert =0. $$

Also, noting that $\Vert x_{k}-Vx_{k} \Vert \leq \Vert x_{k}-V_{k}x_{k} \Vert + \Vert V_{k}x_{k}-Vx_{k} \Vert $, from (32) and (33) we get

36 $$ \lim_{k\to\infty} \Vert x_{k}-Vx_{k} \Vert =0. $$

In addition, observing that

$$\begin{aligned} \Vert x_{k}-Gx_{k} \Vert \leq& \Vert x_{k}-z_{k} \Vert + \Vert z_{k}-Gz_{k} \Vert + \Vert Gz_{k}-Gx_{k} \Vert \\ \leq& \Vert x_{k}-z_{k} \Vert + \Vert z_{k}-Gz_{k} \Vert + \Vert z_{k}-x_{k} \Vert \\ =& 2 \Vert x_{k}-z_{k} \Vert + \Vert z_{k}-Gz_{k} \Vert , \end{aligned}$$

from (31) and (32) we get

37 $$ \lim_{k\to\infty} \Vert x_{k}-Gx_{k} \Vert =0. $$

Since *X* is reflexive, there exists at least a weak convergence subsequence of $\{x_{k}\}$, and hence $\omega_{w}(x_{k})\neq\emptyset$. Take an arbitrary $p\in\omega_{w}(x_{k})$. Then there exists a subsequence $\{x_{k_{i}}\}$ of $\{x_{k}\}$ such that $x_{k_{i}}\rightharpoonup p$. Since $V_{k}$ is nonexpansive for all $k\geq1$, *V* is a nonexpansive self-mapping on *C*. Also, since *X* is uniformly convex and *V* and *G* are two nonexpansive self-mappings on *C*, utilizing Lemma 6 we know from (36) and (37) that $p\in \operatorname{GSVI} (C,A,B)$ and $p\in\operatorname{Fix}(V)=\bigcap^{\infty}_{i=1}\operatorname{Fix}(T_{i})$ (due to Lemma 11). Consequently, $p\in\operatorname{Fix}(V)=\bigcap^{\infty}_{i=1}\operatorname{Fix}(T_{i})\cap\operatorname {GSVI}(C,A,B)=:{\mathcal{F}}$. This shows that $\omega_{w}(x_{k})\subset {\mathcal{F}}$.

*Step 4.* We show that $\omega_{w}(x_{k})=\omega_{s}(x_{k})$, where

$$\omega_{s}(x_{k})=\bigl\{ x\in C:x_{k_{i}}\to x\mbox{ for some subsequence }\{ x_{k_{i}}\}\mbox{ of } \{x_{k}\}\bigr\} . $$

Indeed, by Lemma 9, we have $\Vert GV_{k}x_{k}-z \Vert \leq \Vert x_{k}-z \Vert $ for any fixed $z\in{\mathcal{F}}$, and hence

$$\begin{aligned} \Vert x_{k}-z \Vert ^{2} =& \bigl\Vert \gamma_{k}\Pi_{C}(I-\lambda_{k}F)GV_{k}x_{k}+(1- \gamma_{k})GV_{k}x_{k}-z \bigr\Vert ^{2} \\ =& \gamma_{k}\bigl[\bigl\langle \Pi_{C}(I- \lambda_{k}F)GV_{k}x_{k}-(I-\lambda _{k}F)GV_{k}x_{k},j(x_{k}-z)\bigr\rangle \\ &{} +\bigl\langle \lambda_{k}(I-F)GV_{k}x_{k}+(1- \lambda _{k})GV_{k}x_{k}-z,j(x_{k}-z)\bigr\rangle \bigr]\\ &{}+(1-\gamma_{k})\bigl\langle GV_{k}x_{k}-z,j(x_{k}-z) \bigr\rangle \\ =& \gamma_{k}\bigl[\bigl\langle \Pi_{C}(I- \lambda_{k}F)GV_{k}x_{k}-(I-\lambda _{k}F)GV_{k}x_{k},j\bigl(\Pi_{C}(I- \lambda_{k}F)GV_{k}x_{k}-z\bigr)\bigr\rangle \\ &{} +\bigl\langle \Pi_{C}(I-\lambda_{k}F)GV_{k}x_{k}-(I- \lambda _{k}F)GV_{k}x_{k},j(x_{k}-z)\\ &{}-j \bigl(\Pi_{C}(I-\lambda_{k}F)GV_{k}x_{k}-z \bigr)\bigr\rangle \\ &{} +\bigl\langle \lambda_{k}(I-F)GV_{k}x_{k}+(1- \lambda _{k})GV_{k}x_{k}-z,j(x_{k}-z)\bigr\rangle \bigr]\\ &{}+(1-\gamma_{k})\bigl\langle GV_{k}x_{k}-z,j(x_{k}-z) \bigr\rangle \\ \leq&\gamma_{k}\bigl[ \bigl\Vert \Pi_{C}(I- \lambda_{k}F)GV_{k}x_{k}-(I-\lambda_{k}F)GV_{k}x_{k} \bigr\Vert \bigl\Vert j(x_{k}-z)\\ &{}-j\bigl(\Pi_{C}(I- \lambda_{k}F)GV_{k}x_{k}-z\bigr) \bigr\Vert \\ &{}+\bigl\langle \lambda_{k}(I-F)GV_{k}x_{k}+(1- \lambda _{k})GV_{k}x_{k}-z,j(x_{k}-z)\bigr\rangle \bigr]\\ &{}+(1-\gamma_{k})\bigl\langle GV_{k}x_{k}-z,j(x_{k}-z) \bigr\rangle \\ \leq&\gamma_{k}\bigl[ \bigl\Vert \Pi_{C}(I- \lambda_{k}F)GV_{k}x_{k}-(I-\lambda_{k}F)GV_{k}x_{k} \bigr\Vert \bigl\Vert j(x_{k}-z)\\ &{}-j\bigl(\Pi_{C}(I- \lambda_{k}F)GV_{k}x_{k}-z\bigr) \bigr\Vert \\ &{}+\lambda_{k}\bigl\langle (I-F)GV_{k}x_{k}-z,j(x_{k}-z) \bigr\rangle +(1-\lambda_{k}) \Vert x_{k}-z \Vert ^{2}\bigr]\\ &{}+(1-\gamma_{k}) \Vert x_{k}-z \Vert ^{2} \\ \leq&\gamma_{k}\bigl( \bigl\Vert \Pi_{C}(I- \lambda_{k}F)GV_{k}x_{k}-GV_{k}x_{k} \bigr\Vert \\ &{} +\lambda_{k} \bigl\Vert F(GV_{k}x_{k}) \bigr\Vert \bigr) \bigl\Vert j(x_{k}-z)-j\bigl(\Pi_{C}(I- \lambda_{k}F)GV_{k}x_{k}-z\bigr) \bigr\Vert \\ &{} +\gamma_{k}\lambda_{k}\bigl\langle (I-F)GV_{k}x_{k}-z,j(x_{k}-z) \bigr\rangle +(1-\gamma _{k}\lambda_{k}) \Vert x_{k}-z \Vert ^{2} \\ \leq& 2\gamma_{k}\lambda_{k} \bigl\Vert F(GV_{k}x_{k}) \bigr\Vert \bigl\Vert j(x_{k}-z)-j \bigl(\Pi_{C}(I-\lambda_{k}F)GV_{k}x_{k}-z \bigr) \bigr\Vert \\ &{} +\gamma_{k}\lambda_{k}\bigl\langle (I-F)GV_{k}x_{k}-(I-F)z-F(z),j(x_{k}-z) \bigr\rangle +(1-\gamma_{k}\lambda_{k}) \Vert x_{k}-z \Vert ^{2}. \end{aligned}$$

Therefore, by Lemma 8(b) we get

$$\begin{aligned} \Vert x_{k}-z \Vert ^{2} \leq& (1-\tau) \Vert x_{k}-z \Vert ^{2}-\bigl\langle F(z),j(x_{k}-z) \bigr\rangle \\ &{} +2 \bigl\Vert F(x_{k}) \bigr\Vert \bigl\Vert j(x_{k}-z)-j\bigl(\Pi_{C}(I-\lambda_{k}F)x_{k}-z \bigr) \bigr\Vert , \end{aligned}$$

which immediately leads to

38 $$\begin{aligned} \Vert x_{k}-z \Vert ^{2} \leq{}&\frac{1}{\tau}\bigl(\bigl\langle F(z),j(z-x_{k})\bigr\rangle \\ &{}+2 \bigl\Vert F(GV_{k}x_{k}) \bigr\Vert \bigl\Vert j(x_{k}-z)-j\bigl(\Pi_{C}(I-\lambda_{k}F)GV_{k}x_{k}-z \bigr) \bigr\Vert \bigr),\quad\forall z\in{\mathcal{F}}, \end{aligned}$$

where $\tau=1-\sqrt{\frac{1-\delta}{\zeta}}\in(0,1)$. Note that

$$\begin{aligned} \bigl\Vert (x_{k}-z)-\bigl(\Pi_{C}(I- \lambda_{k}F)GV_{k}x_{k}-z\bigr) \bigr\Vert &\leq \bigl\Vert x_{k}-(I-\lambda_{k}F)y_{k} \bigr\Vert \\ &\leq \Vert x_{k}-y_{k} \Vert + \lambda_{k} \bigl\Vert F(y_{k}) \bigr\Vert . \end{aligned}$$

Since the uniform smoothness of *X* guarantees the uniform continuity of *j* on every nonempty bounded subset of *X*, we deduce from (32), $\lambda_{k}\to0$ and the boundedness of $\{y_{k}\}$ that

$$\lim_{k\to\infty} \bigl\Vert j(x_{k}-z)-j\bigl( \Pi_{C}(I-\lambda_{k}F)GV_{k}x_{k}-z \bigr) \bigr\Vert =0. $$

Now, take an arbitrary $p\in\omega_{w}(x_{k})$. Then there exists a subsequence $\{x_{k_{i}}\}$ of $\{x_{k}\}$ such that $x_{k_{i}} \rightharpoonup p$. In terms of Step 3, we know that $p\in\omega _{w}(x_{k})\subset{\mathcal{F}}$. Thus, we can substitute $x_{k_{i}}$ for $x_{k}$ and *p* for *z* in (38) to get

39 $$\begin{aligned} \Vert x_{k_{i}}-p \Vert ^{2} \leq{}&\frac{1}{\tau}\bigl(\bigl\langle F(p),j(p-x_{k_{i}})\bigr\rangle \\ &{}+2 \bigl\Vert F(y_{k_{i}}) \bigr\Vert \bigl\Vert j(x_{k_{i}}-p)-j\bigl(\Pi_{C}(I-\lambda_{k_{i}}F)y_{k_{i}}-p \bigr) \bigr\Vert \bigr). \end{aligned}$$

Consequently, the weak convergence of $\{x_{k_{i}}\}$ to *p* together with (39) actually implies that $x_{k_{i}}\to p$ as $i\to\infty $, and hence $p\in\omega_{s}(x_{k})$. This shows that $\omega_{w}(x_{k})=\omega_{s}(x_{k})$.

*Step 5.* We show that each $p\in\omega_{s}(x_{k})$ solves the variational inequality (19). Indeed, take an arbitrary $p\in\omega_{s}(x_{k})$. Then there exists a subsequence $\{x_{k_{i}}\}$ of $\{x_{k}\}$ such that $x_{k_{i}}\to p$ as $i\to\infty$. According to Steps 3 and 4, we know that $p\in\omega_{s}(x_{k})$ $(=\omega _{w}(x_{k})\subset{\mathcal{F}})$. Replacing $x_{k}$ in (39) with $x_{k_{i}}$, and noticing that $x_{k_{i}}\to p$, we have the Minty type variational inequality

$$\bigl\langle F(z),j(z-p)\bigr\rangle \leq0,\quad\forall z\in{\mathcal{F}}, $$

which is equivalent to the variational inequality (see Lemma 7)

40 $$ \bigl\langle F(p),j(p-z)\bigr\rangle \leq0,\quad\forall z\in{ \mathcal{F}}. $$

That is, $p\in{\mathcal{F}}$ is a solution of (19).

*Step 6.* We show that $\{x_{k}\}$ converges strongly to a unique solution in ${\mathcal{F}}$ to VI (19). Indeed, we first claim that the solution set of (19) is a singleton. As a matter of fact, assume that $\bar{p}\in{\mathcal{F}}$ is also a solution of (19). Then we have

$$\bigl\langle F(\bar{p}),j(\bar{p}-p)\bigr\rangle \leq0. $$

From (40), we have

$$\bigl\langle F(p),j(p-\bar{p})\bigr\rangle \leq0. $$

So, by the *δ*-strong accretiveness of *F*, we have

$$\begin{aligned} &\bigl\langle F(\bar{p}),j(\bar{p}-p)\bigr\rangle +\bigl\langle F(p),j(p-\bar{p}) \bigr\rangle \leq0 \\ &\quad\Rightarrow\bigl\langle F(\bar{p})-F(p),j(\bar{p}-p)\bigr\rangle \leq0 \\ &\quad\Rightarrow\delta \Vert \bar{p}-p \Vert ^{2}\leq0. \end{aligned}$$

Therefore, $\bar{p}=p$. In summary, we have shown that each cluster point of $\{x_{k}\}$ (as $k\to\infty$) equals *p*. Consequently, $x_{k}\to p$ as $k\to\infty$. □

### Theorem 6

*Let* $C,X,\Pi_{C},A,B,F,\{T_{i}\}^{\infty}_{i=1},{\mathcal{F}},\delta,\zeta,\lambda$ *and* *μ* *be as in Theorem* 5. *Let* $\{V_{k}\}^{\infty}_{k=1}$ *be defined by* (15) *and* (16). *For arbitrarily given* $x_{1}\in C$, *let* $\{x_{k}\}^{\infty}_{k=1}$ *be defined by*

$$\begin{aligned} x_{k+1} =&\beta_{k}x_{k}+\gamma_{k} \Pi_{C}(I-\lambda A)\Pi_{C}(I-\mu B)V_{k}x_{k} \\ &{}+(1-\beta_{k}-\gamma_{k})\Pi_{C}(I- \lambda_{k}F)\Pi_{C}(I-\lambda A)\Pi _{C}(I-\mu B)V_{k}x_{k},\quad\forall k\geq1, \end{aligned}$$

*where* $\{\lambda_{k}\},\{\beta_{k}\}$ *and* $\{\gamma_{k}\}$ *are three sequences in* $[0,1]$ *with* $\beta_{k}+\gamma_{k}\leq1,\forall k\geq1$, *and satisfy the following conditions*:

(i) $\lim_{k\to\infty}\lambda_{k}=0$ *and* $\sum^{\infty}_{k=1}\lambda _{k}=\infty$;
(ii) $\lim_{k\to\infty}(\frac{\gamma_{k+1}}{1-\beta_{k+1}}-\frac {\gamma_{k}}{1-\beta_{k}})=0$;
(iii) $0<\liminf_{k\to\infty}\beta_{k}\leq\limsup_{k\to\infty }(\beta_{k}+\gamma_{k})<1$.

*Then* $\{x_{k}\}^{\infty}_{k=1}$ *converges strongly to a unique solution* $x^{*}\in{\mathcal{F}}$.

### Proof

Let the mapping $G:C\to C$ be defined as $G:=\Pi _{C}(I-\lambda A)\Pi_{C}(I-\mu B)$, where $0<\lambda<\frac {\alpha}{\kappa^{2}}$ and $0<\mu<\frac{\beta}{\kappa^{2}}$. In terms of Lemma 4 we know that $G:C\to C$ is nonexpansive. Then the explicit iterative scheme can be rewritten as

41 $$ x_{k+1}=\beta_{k}x_{k}+ \gamma_{k}GV_{k}x_{k}+(1-\beta_{k}- \gamma_{k})\Pi_{C}(I-\lambda _{k}F)GV_{k}x_{k}, \quad\forall k\geq1. $$

Next, we divide the rest of the proof into several steps.

*Step 1.* We show that $\{x_{k}\}^{\infty}_{k=1}$ is bounded. Indeed, take an arbitrarily given $p\in{\mathcal{F}}$. Then we have $V_{k}p=p$ and $Gp=p$. Hence, by Lemma 8(c) we get

$$\begin{aligned} \Vert x_{k+1}-p \Vert =& \bigl\Vert \beta_{k}x_{k}+ \gamma_{k}GV_{k}x_{k}+(1-\beta_{k}- \gamma_{k})\Pi_{C}(I-\lambda_{k}F)GV_{k}x_{k}-p \bigr\Vert \\ \leq&\beta_{k} \Vert x_{k}-p \Vert + \gamma_{k} \Vert GV_{k}x_{k}-p \Vert +(1- \beta_{k}-\gamma_{k}) \bigl\Vert \Pi_{C}(I- \lambda_{k}F)GV_{k}x_{k}-p \bigr\Vert \\ \leq&\beta_{k} \Vert x_{k}-p \Vert + \gamma_{k} \Vert x_{k}-p \Vert \\ &{}+(1-\beta_{k}-\gamma_{k}) \bigl\Vert (I- \lambda_{k}F)GV_{k}x_{k}-(I-\lambda_{k}F)p- \lambda_{k}F(p) \bigr\Vert \\ \leq& (\beta_{k}+\gamma_{k}) \Vert x_{k}-p \Vert +(1-\beta_{k}-\gamma_{k}) \bigl\Vert (I- \lambda_{k}F)GV_{k}x_{k}-(I-\lambda_{k}F)p \bigr\Vert \\ &{} +\lambda_{k}(1-\beta_{k}-\gamma_{k}) \bigl\Vert F(p) \bigr\Vert \\ \leq& (\beta_{k}+\gamma_{k}) \Vert x_{k}-p \Vert +(1-\beta_{k}-\gamma_{k}) (1-\lambda_{k}\tau ) \Vert GV_{k}x_{k}-p \Vert \\ &{}+\lambda_{k}(1- \beta_{k}-\gamma_{k}) \bigl\Vert F(p) \bigr\Vert \\ \leq& \bigl[1-(1-\beta_{k}-\gamma_{k})\lambda_{k} \tau\bigr] \Vert x_{k}-p \Vert +(1-\beta_{k}-\gamma _{k})\lambda_{k}\tau\frac{ \Vert F(p) \Vert }{\tau}. \end{aligned}$$

By induction, we conclude that

$$\Vert x_{k+1}-p \Vert \leq\max\biggl\{ \Vert x_{0}-p \Vert ,\frac{ \Vert F(p) \Vert }{\tau}\biggr\} . $$

Therefore, $\{x_{k}\}^{\infty}_{k=1}$ is bounded. So, the sequences $\{z_{k}\} ^{\infty}_{k=1}$, $\{y_{k}\}^{\infty}_{k=1}$ and $\{F(y_{k})\}^{\infty}_{k=1}$, where $z_{k}=V_{k}x_{k}$ and $y_{k}=GV_{k}x_{k}$, are also bounded.

*Step 2.* We show that $\Vert x_{k+1}-x_{k} \Vert \to0$ and $\Vert x_{k}-y_{k} \Vert \to0$ as $k\to\infty$. Indeed, observe that

$$\begin{aligned} \Vert y_{k+1}-y_{k} \Vert =& \Vert GV_{k+1}x_{k+1}-GV_{k}x_{k} \Vert \\ \leq& \Vert V_{k+1}x_{k+1}-V_{k}x_{k} \Vert \\ \leq& \Vert V_{k+1}x_{k+1}-V_{k+1}x_{k} \Vert + \Vert V_{k+1}x_{k}-V_{k}x_{k} \Vert \\ \leq& \Vert x_{k+1}-x_{k} \Vert + \bigl\Vert V_{k}T^{k+1}x_{k}-V_{k}x_{k} \bigr\Vert \\ \leq& \Vert x_{k+1}-x_{k} \Vert + \bigl\Vert T^{k+1}x_{k}-x_{k} \bigr\Vert \\ =& \Vert x_{k+1}-x_{k} \Vert +\alpha_{k+1} \Vert x_{k}-T_{k+1}x_{k} \Vert . \end{aligned}$$

Set $x_{k+1}=\beta_{k}x_{k}+(1-\beta_{k})w_{k}$ for all $k\geq1$. Then $w_{k}=\frac{\gamma_{k}y_{k}+(1-\beta_{k}-\gamma_{k})\Pi_{C}(I-\lambda _{k}F)y_{k}}{1-\beta_{k}}$. Note that

$$\begin{aligned} & \bigl\Vert \Pi_{C}(I-\lambda_{k+1}F)y_{k+1}- \Pi_{C}(I-\lambda_{k}F)y_{k} \bigr\Vert \\ &\quad\leq \bigl\Vert (I-\lambda_{k+1}F)y_{k+1}-(I- \lambda_{k}F)y_{k} \bigr\Vert \\ &\quad= \bigl\Vert y_{k+1}-y_{k}-\lambda_{k+1}F(y_{k+1})+ \lambda_{k}F(y_{k}) \bigr\Vert \\ &\quad\leq \Vert y_{k+1}-y_{k} \Vert + \lambda_{k+1} \bigl\Vert F(y_{k+1}) \bigr\Vert + \lambda_{k} \bigl\Vert F(y_{k}) \bigr\Vert \\ &\quad\leq \Vert x_{k+1}-x_{k} \Vert + \lambda_{k+1} \bigl\Vert F(y_{k+1}) \bigr\Vert + \lambda_{k} \bigl\Vert F(y_{k}) \bigr\Vert + \alpha_{k+1} \Vert x_{k}-T_{k+1}x_{k} \Vert . \end{aligned}$$

Hence

$$\begin{aligned} & \Vert w_{k+1}-w_{k} \Vert \\ &\quad= \biggl\Vert \frac{\gamma_{k+1}y_{k+1}+(1-\beta_{k+1}-\gamma_{k+1})\Pi_{C}(I-\lambda_{k+1}F)y_{k+1}}{1-\beta_{k+1}} \\ &\qquad {}-\frac{\gamma_{k}y_{k}+(1-\beta_{k}-\gamma_{k})\Pi_{C}(I-\lambda _{k}F)y_{k}}{1-\beta_{k}} \biggr\Vert \\ &\quad\leq \biggl\Vert \frac{\gamma_{k+1}}{1-\beta_{k+1}}y_{k+1}- \frac{\gamma _{k}}{1-\beta_{k}}y_{k} \biggr\Vert \\ &\qquad{} + \biggl\Vert \frac{(1-\beta_{k+1}-\gamma_{k+1})}{1-\beta_{k+1}}\Pi_{C}(I- \lambda_{k+1}F)y_{k+1}-\frac{(1-\beta_{k}-\gamma_{k})}{1-\beta_{k}}\Pi_{C}(I- \lambda_{k}F)y_{k} \biggr\Vert \\ &\quad\leq \biggl\vert \frac{\gamma_{k+1}}{1-\beta_{k+1}}-\frac{\gamma_{k}}{1-\beta _{k}} \biggr\vert \Vert y_{k+1} \Vert +\frac{\gamma_{k}}{1-\beta_{k}}| \Vert y_{k+1}-y_{k} \Vert \\ &\qquad{} + \biggl\vert \frac{(1-\beta_{k+1}-\gamma_{k+1})}{1-\beta_{k+1}}-\frac {(1-\beta_{k}-\gamma_{k})}{1-\beta_{k}} \biggr\vert \bigl\Vert \Pi_{C}(I-\lambda_{k+1}F)y_{k+1} \bigr\Vert \\ &\qquad{}+\frac{(1-\beta_{k}-\gamma_{k})}{1-\beta_{k}} \bigl\Vert \Pi_{C}(I-\lambda _{k+1}F)y_{k+1}-\Pi_{C}(I-\lambda_{k}F)y_{k} \bigr\Vert \\ &\quad\leq \biggl\vert \frac{\gamma_{k+1}}{1-\beta_{k+1}}-\frac{\gamma_{k}}{1-\beta _{k}} \biggr\vert \bigl( \Vert y_{k+1} \Vert + \bigl\Vert \Pi_{C}(I- \lambda_{k+1}F)y_{k+1} \bigr\Vert \bigr) \\ &\qquad{} +\frac{\gamma_{k}}{1-\beta_{k}}|\bigl( \Vert x_{k+1}-x_{k} \Vert +\alpha_{k+1} \Vert x_{k}-T_{k+1}x_{k} \Vert \bigr) \\ &\qquad{} +\frac{(1-\beta_{k}-\gamma_{k})}{1-\beta_{k}}\bigl( \Vert x_{k+1}-x_{k} \Vert +\lambda_{k+1} \bigl\Vert F(y_{k+1}) \bigr\Vert + \lambda_{k} \bigl\Vert F(y_{k}) \bigr\Vert + \alpha_{k+1} \Vert x_{k}-T_{k+1}x_{k} \Vert \bigr) \\ &\quad\leq \biggl\vert \frac{\gamma_{k+1}}{1-\beta_{k+1}}-\frac{\gamma_{k}}{1-\beta _{k}} \biggr\vert \bigl( \Vert y_{k+1} \Vert + \bigl\Vert \Pi_{C}(I- \lambda_{k+1}F)y_{k+1} \bigr\Vert \bigr) \\ &\qquad{} + \Vert x_{k+1}-x_{k} \Vert + \lambda_{k+1} \bigl\Vert F(y_{k+1}) \bigr\Vert + \lambda_{k} \bigl\Vert F(y_{k}) \bigr\Vert + \alpha_{k+1}\bigl( \Vert x_{k} \Vert + \Vert T_{k+1}x_{k} \Vert \bigr). \end{aligned}$$

Since $\{x_{k}\},\{y_{k}\}$ and $\{F(y_{k})\}$ are bounded, we have that $\{ \Vert y_{k+1} \Vert + \Vert \Pi_{C}(I-\lambda_{k+1}F)y_{k+1} \Vert \}$ and $\{ \Vert x_{k} \Vert + \Vert T_{k+1}x_{k} \Vert \}$ are bounded. So it follows from $\alpha _{k}\to0$ and conditions (i) and (ii) that

$$\limsup_{k\to\infty}\bigl( \Vert w_{k+1}-w_{k} \Vert - \Vert x_{k+1}-x_{k} \Vert \bigr)\leq0. $$

Hence, by Lemma 12, we get $\Vert w_{k}-x_{k} \Vert \to0$ as $k\to\infty$. Consequently,

42 $$ \lim_{k\to\infty} \Vert x_{k+1}-x_{k} \Vert =\lim_{k\to\infty}(1-\beta_{k}) \Vert w_{k}-x_{k} \Vert =0. $$

We also note that

$$\begin{aligned} \Vert w_{k}-y_{k} \Vert =& \biggl\Vert \frac{\gamma_{k}y_{k}+(1-\beta_{k}-\gamma_{k})\Pi_{C}(I-\lambda_{k}F)y_{k}}{1-\beta_{k}}-y_{k} \biggr\Vert \\ =&\frac{1-\beta_{k}-\gamma_{k}}{1-\beta_{k}} \bigl\Vert \Pi_{C}(I- \lambda_{k}F)y_{k}-y_{k} \bigr\Vert \\ \leq& \bigl\Vert \Pi_{C}(I-\lambda_{k}F)y_{k}- \Pi_{C}y_{k} \bigr\Vert \\ \leq&\lambda_{k} \bigl\Vert F(y_{k}) \bigr\Vert \to0 \quad\mbox{as } k\to\infty. \end{aligned}$$

It follows that

43 $$ \lim_{k\to\infty} \Vert x_{k}-y_{k} \Vert =0. $$

*Step 3.* We show that $\Vert z_{k}-Gz_{k} \Vert \to0$ and $\Vert x_{k}-V_{k}x_{k} \Vert \to0$ as $k\to\infty$. Indeed, for simplicity, put $q=\Pi_{C}(p-\mu Bp)$ and $u_{k}=\Pi _{C}(z_{k}-\mu Bz_{k})$. Then $y_{k}=Gz_{k}=\Pi_{C}(u_{k}- \lambda Au_{k})$ for all $k\geq1$. Repeating the same arguments as those of (24), we obtain

44 $$ \Vert y_{k}-p \Vert ^{2}\leq \Vert x_{k}-p \Vert ^{2}-2\mu\bigl(\beta -\kappa^{2}\mu \bigr) \Vert Bz_{k}-Bp \Vert ^{2}-2\lambda\bigl(\alpha- \kappa^{2}\lambda\bigr) \Vert Au_{k}-Aq \Vert ^{2}. $$

Observe that

45 $$\begin{aligned} \Vert x_{k+1}-p \Vert ^{2} \leq{}&\beta_{k} \Vert x_{k}-p \Vert ^{2}+ \gamma_{k} \Vert GV_{k}x_{k}-p \Vert ^{2}+(1-\beta_{k}-\gamma _{k}) \bigl\Vert \Pi_{C}(I-\lambda_{k}F)GV_{k}x_{k}-p \bigr\Vert ^{2} \\ \leq{}&\beta_{k} \Vert x_{k}-p \Vert ^{2}+ \gamma_{k} \Vert GV_{k}x_{k}-p \Vert ^{2} \\ &{}+(1-\beta_{k}-\gamma_{k}) \bigl\Vert (I- \lambda_{k}F)GV_{k}x_{k}-(I-\lambda_{k}F)p- \lambda_{k}F(p) \bigr\Vert ^{2} \\ \leq{}&\beta_{k} \Vert x_{k}-p \Vert ^{2}+ \gamma_{k} \Vert GV_{k}x_{k}-p \Vert ^{2} \\ &{}+(1-\beta_{k}-\gamma_{k})\bigl[(1- \lambda_{k}\tau) \Vert GV_{k}x_{k}-p \Vert + \lambda_{k}F(p)\|\bigr]^{2} \\ \leq{}&\beta_{k} \Vert x_{k}-p \Vert ^{2}+ \gamma_{k} \Vert GV_{k}x_{k}-p \Vert ^{2} \\ &{}+(1-\beta_{k}-\gamma_{k})\bigl[(1- \lambda_{k}\tau) \Vert GV_{k}x_{k}-p \Vert ^{2}+\lambda _{k}\tau^{-1} \bigl\Vert F(p) \bigr\Vert ^{2}\bigr] \\ \leq{}&\beta_{k} \Vert x_{k}-p \Vert ^{2}+ \gamma_{k} \Vert GV_{k}x_{k}-p \Vert ^{2} \\ &{}+(1-\beta_{k}-\gamma_{k}) \Vert GV_{k}x_{k}-p \Vert ^{2}+\lambda_{k} \tau^{-1} \bigl\Vert F(p) \bigr\Vert ^{2} \\ ={}&\beta_{k} \Vert x_{k}-p \Vert ^{2}+(1- \beta_{k}) \Vert GV_{k}x_{k}-p \Vert ^{2}+\lambda_{k}\tau ^{-1} \bigl\Vert F(p) \bigr\Vert ^{2}, \end{aligned}$$

which together with (44) implies that

$$\begin{aligned} \Vert x_{k+1}-p \Vert ^{2} \leq{}& \beta_{k} \Vert x_{k}-p \Vert ^{2}+(1- \beta_{k})\bigl[ \Vert x_{k}-p \Vert ^{2}-2\mu \bigl(\beta-\kappa ^{2}\mu\bigr) \Vert Bz_{k}-Bp \Vert ^{2} \\ &{}-2\lambda\bigl(\alpha-\kappa^{2}\lambda\bigr) \Vert Au_{k}-Aq \Vert ^{2}\bigr]+\lambda_{k}\tau ^{-1} \bigl\Vert F(p) \bigr\Vert ^{2} \\ ={}& \Vert x_{k}-p \Vert ^{2}-2(1- \beta_{k})\bigl[\mu\bigl(\beta-\kappa^{2}\mu\bigr) \Vert Bz_{k}-Bp \Vert ^{2} \\ &{}+\lambda\bigl(\alpha-\kappa^{2}\lambda\bigr) \Vert Au_{k}-Aq \Vert ^{2}\bigr]+\lambda_{k}\tau ^{-1} \bigl\Vert F(p) \bigr\Vert ^{2}. \end{aligned}$$

So, it follows that

46 $$\begin{aligned} &2(1-\beta_{k})\bigl[\mu\bigl(\beta-\kappa^{2} \mu\bigr) \Vert Bz_{k}-Bp \Vert ^{2}+\lambda\bigl(\alpha- \kappa ^{2}\lambda\bigr) \Vert Au_{k}-Aq \Vert ^{2}\bigr] \\ &\quad\leq \Vert x_{k}-p \Vert ^{2}- \Vert x_{k+1}-p \Vert ^{2}+\lambda_{k} \tau^{-1} \bigl\Vert F(p) \bigr\Vert ^{2} \\ &\quad\leq \Vert x_{k}-x_{k+1} \Vert \bigl( \Vert x_{k}-p \Vert + \Vert x_{k+1}-p \Vert \bigr)+ \lambda_{k}\tau ^{-1} \bigl\Vert F(p) \bigr\Vert ^{2}. \end{aligned}$$

Since $\lambda\in(0,\frac{\alpha}{\kappa^{2}})$, $\mu\in(0,\frac{\beta }{\kappa^{2}})$ and $\lambda_{k}\to0$ as $k\to\infty$, we deduce from (42) and condition (iii) that

47 $$ \lim_{k\to\infty} \Vert Bz_{k}-Bp \Vert =0\quad\mbox{and}\quad\lim_{k\to\infty} \Vert Au_{k}-Aq \Vert =0. $$

Repeating the same arguments as those of (28), we get

48 $$\begin{aligned} \Vert y_{k}-p \Vert ^{2} \leq& \Vert x_{k}-p \Vert ^{2}-g_{1}\bigl( \bigl\Vert z_{k}-u_{k}-(p-q) \bigr\Vert \bigr)-g_{2}\bigl( \bigl\Vert u_{k}-y_{k}+(p-q) \bigr\Vert \bigr) \\ &{}+2\mu \Vert Bp-Bz_{k} \Vert \Vert u_{k}-q \Vert +2\lambda \Vert Aq-Au_{k} \Vert \Vert y_{k}-p \Vert . \end{aligned}$$

Combining (45) and (48), we have

$$\begin{aligned} \Vert x_{k+1}-p \Vert ^{2} \leq&\beta_{k} \Vert x_{k}-p \Vert ^{2}+(1-\beta_{k})\bigl[ \Vert x_{k}-p \Vert ^{2}-g_{1}\bigl( \bigl\Vert z_{k}-u_{k}-(p-q) \bigr\Vert \bigr) \\ &{}-g_{2}\bigl( \bigl\Vert u_{k}-y_{k}+(p-q) \bigr\Vert \bigr)+2\mu \Vert Bp-Bz_{k} \Vert \Vert u_{k}-q \Vert \\ &+2\lambda \Vert Aq-Au_{k} \Vert \Vert y_{k}-p \Vert \bigr]+\lambda_{k}\tau^{-1} \bigl\Vert F(p) \bigr\Vert ^{2} \\ \leq& \Vert x_{k}-p \Vert ^{2}-(1-\beta_{k}) \bigl[g_{1}\bigl( \bigl\Vert z_{k}-u_{k}-(p-q) \bigr\Vert \bigr)+g_{2}\bigl( \bigl\Vert u_{k}-y_{k}+(p-q) \bigr\Vert \bigr)\bigr] \\ &{}+2\mu \Vert Bp-Bz_{k} \Vert \Vert u_{k}-q \Vert +2\lambda \Vert Aq-Au_{k} \Vert \Vert y_{k}-p \Vert + \lambda_{k}\tau ^{-1} \bigl\Vert F(p) \bigr\Vert ^{2}, \end{aligned}$$

which immediately leads to

$$\begin{aligned} &(1-\beta_{k})\bigl[g_{1}\bigl( \bigl\Vert z_{k}-u_{k}-(p-q) \bigr\Vert \bigr)+g_{2}\bigl( \bigl\Vert u_{k}-y_{k}+(p-q) \bigr\Vert \bigr)\bigr] \\ &\quad\leq \Vert x_{k}-p \Vert ^{2}- \Vert x_{k+1}-p \Vert ^{2} \\ &\qquad{}+2\mu \Vert Bp-Bz_{k} \Vert \Vert u_{k}-q \Vert +2\lambda \Vert Aq-Au_{k} \Vert \Vert y_{k}-p \Vert +\lambda _{k}\tau^{-1} \bigl\Vert F(p) \bigr\Vert ^{2} \\ &\quad\leq \Vert x_{k}-x_{k+1} \Vert \bigl( \Vert x_{k}-p \Vert + \Vert x_{k+1}-p \Vert \bigr) \\ &\qquad{}+2\mu \Vert Bp-Bz_{k} \Vert \Vert u_{k}-q \Vert +2\lambda \Vert Aq-Au_{k} \Vert \Vert y_{k}-p \Vert +\lambda _{k}\tau^{-1} \bigl\Vert F(p) \bigr\Vert ^{2}. \end{aligned}$$

Since $\lambda_{k}\to0$ as $k\to\infty$, and $\{u_{k}\}$ and $\{y_{k}\}$ are bounded, we deduce from (42), (47) and condition (iii) that

$$\lim_{k\to\infty}g_{1}\bigl( \bigl\Vert z_{k}-u_{k}-(p-q) \bigr\Vert \bigr)=0\quad\mbox{and}\quad \lim_{k\to \infty}g_{2}\bigl( \bigl\Vert u_{k}-y_{k}+(p-q) \bigr\Vert \bigr)=0. $$

Utilizing the properties of $g_{1}$ and $g_{2}$, we conclude that

49 $$ \lim_{k\to\infty} \bigl\Vert z_{k}-u_{k}-(p-q) \bigr\Vert =0\quad\mbox{and}\quad\lim_{k\to\infty } \bigl\Vert u_{k}-y_{k}+(p-q) \bigr\Vert =0. $$

From (49), we get

$$\Vert z_{k}-y_{k} \Vert \leq \bigl\Vert z_{k}-u_{k}-(p-q) \bigr\Vert + \bigl\Vert u_{k}-y_{k}+(p-q) \bigr\Vert \to0\quad\mbox{as }k\to\infty. $$

That is,

50 $$ \lim_{k\to\infty} \Vert z_{k}-Gz_{k} \Vert =\lim_{k\to\infty} \Vert z_{k}-y_{k} \Vert =0. $$

This together with (43) implies that

51 $$ \lim_{k\to\infty} \Vert x_{k}-V_{k}x_{k} \Vert =\lim_{k\to\infty} \Vert x_{k}-z_{k} \Vert =0. $$

*Step 4.* We show that $\omega_{w}(x_{k})\subset{\mathcal{F}}$, where

$$\omega_{w}(x_{k})=\bigl\{ x\in C:x_{k_{i}} \rightharpoonup x\mbox{ for some subsequence }\{x_{k_{i}}\}\mbox{ of } \{x_{k}\}\bigr\} . $$

Indeed, repeating the same arguments as those of (36) and (37) in the proof of Theorem 5, we obtain that

52 $$ \lim_{k\to\infty} \Vert x_{k}-Vx_{k} \Vert =0\quad\mbox{and}\quad\lim_{k\to\infty} \Vert x_{k}-Gx_{k} \Vert =0. $$

Utilizing Lemma 6 and Lemma 11 and the nonexpansivity of *V* and *G*, we conclude that $\omega_{w}(x_{k})\subset {\mathcal{F}}$.

*Step 5.* We show that $\limsup_{k\to\infty}\langle F(x^{*}),j(x^{*}-v_{k})\rangle\leq0$, where $v_{k}=\Pi_{C}(I-\lambda_{k}F)y_{k}$ for all $k\geq1$ and $x^{*}\in {\mathcal{F}}$ is the unique solution of VI (19). Indeed, we first take a subsequence $\{v_{k_{i}}\}$ of $\{v_{k}\}$ such that

$$\limsup_{k\to\infty}\bigl\langle F\bigl(x^{*}\bigr),j \bigl(x^{*}-v_{k}\bigr)\bigr\rangle =\lim_{i\to\infty }\bigl\langle F\bigl(x^{*}\bigr),j\bigl(x^{*}-v_{k_{i}}\bigr)\bigr\rangle . $$

We may also assume that $v_{k_{i}}\rightharpoonup z$. Note that

53 $$\begin{aligned} \Vert v_{k}-x_{k} \Vert &= \bigl\Vert \Pi_{C}(I-\lambda_{k}F)y_{k}-x_{k} \bigr\Vert \\ &\leq \bigl\Vert (I-\lambda_{k}F)y_{k}-x_{k} \bigr\Vert \leq \Vert y_{k}-x_{k} \Vert +\lambda_{k} \bigl\Vert F(y_{k}) \bigr\Vert \to0\quad\mbox{as } k\to\infty. \end{aligned}$$

Combining (53) with $\omega_{w}(x_{k})\subset{\mathcal{F}}$ (due to Step 4), we get $z\in{\mathcal{F}}$. So, it follows from VI (19) that

$$\begin{aligned} \limsup_{k\to\infty}\bigl\langle F\bigl(x^{*}\bigr),j \bigl(x^{*}-v_{k}\bigr)\bigr\rangle &=\lim_{i\to\infty }\bigl\langle F\bigl(x^{*}\bigr),j\bigl(x^{*}-v_{k_{i}}\bigr)\bigr\rangle \\ &=\bigl\langle F\bigl(x^{*}\bigr),j\bigl(x^{*}-z\bigr)\bigr\rangle \leq0. \end{aligned}$$

Since $v_{k}=\Pi_{C}(I-\lambda_{k}F)y_{k}$ for all $k\geq1$, according to Lemma 2(iii), we have

54 $$ \bigl\langle (I-\lambda_{k}F)y_{k}- \Pi_{C}(I-\lambda_{k}F)y_{k},j\bigl(x^{*}-v_{k} \bigr)\bigr\rangle \leq0. $$

From (54), we have

$$\begin{aligned} \bigl\Vert v_{k}-x^{*} \bigr\Vert ^{2} =&\bigl\langle \Pi_{C}(I-\lambda_{k}F)y_{k}-x^{*},j \bigl(v_{k}-x^{*}\bigr)\bigr\rangle \\ =&\bigl\langle \Pi_{C}(I-\lambda_{k}F)y_{k}-(I- \lambda_{k}F)y_{k},j\bigl(v_{k}-x^{*}\bigr)\bigr\rangle \\ &{}+\bigl\langle (I-\lambda_{k}F)y_{k}-x^{*},j \bigl(v_{k}-x^{*}\bigr)\bigr\rangle \\ \leq&\bigl\langle (I-\lambda_{k}F)y_{k}-x^{*},j \bigl(v_{k}-x^{*}\bigr)\bigr\rangle \\ =&\bigl\langle (I-\lambda_{k}F)y_{k}-(I- \lambda_{k}F)x^{*},j\bigl(v_{k}-x^{*}\bigr)\bigr\rangle +\lambda _{k}\bigl\langle F\bigl(x^{*}\bigr),j\bigl(x^{*}-v_{k}\bigr)\bigr\rangle \\ \leq&(1-\lambda_{k}\tau) \bigl\Vert y_{k}-x^{*} \bigr\Vert \bigl\Vert v_{k}-x^{*} \bigr\Vert +\lambda_{k}\bigl\langle F\bigl(x^{*}\bigr),j\bigl(x^{*}-v_{k}\bigr)\bigr\rangle \\ \leq&\frac{1}{2}(1-\lambda_{k}\tau)^{2} \bigl\Vert y_{k}-x^{*} \bigr\Vert ^{2}+\frac{1}{2} \bigl\Vert v_{k}-x^{*} \bigr\Vert ^{2}+\lambda_{k}\bigl\langle F\bigl(x^{*}\bigr),j\bigl(x^{*}-v_{k}\bigr)\bigr\rangle , \end{aligned}$$

which immediately yields

55 $$\begin{aligned} \bigl\Vert v_{k}-x^{*} \bigr\Vert ^{2} \leq&(1-\lambda_{k}\tau) \bigl\Vert y_{k}-x^{*} \bigr\Vert ^{2}+2\lambda_{k}\bigl\langle F\bigl(x^{*}\bigr),j \bigl(x^{*}-v_{k}\bigr)\bigr\rangle \\ \leq&(1-\lambda_{k}\tau) \bigl\Vert x_{k}-x^{*} \bigr\Vert ^{2}+2\lambda_{k}\bigl\langle F\bigl(x^{*}\bigr),j \bigl(x^{*}-v_{k}\bigr)\bigr\rangle . \end{aligned}$$

*Step 6.* We show that $x_{k}\to x^{*}$ as $k\to\infty$. Indeed, from (45) and (55), we have

56 $$\begin{aligned} \bigl\Vert x_{k+1}-x^{*} \bigr\Vert ^{2} \leq&\beta_{k} \bigl\Vert x_{k}-x^{*} \bigr\Vert ^{2}+\gamma_{k} \bigl\Vert y_{k}-x^{*} \bigr\Vert ^{2}+(1-\beta_{k}-\gamma_{k}) \bigl\Vert v_{k}-x^{*} \bigr\Vert ^{2} \\ \leq&\beta_{k} \bigl\Vert x_{k}-x^{*} \bigr\Vert ^{2}+\gamma_{k} \bigl\Vert x_{k}-x^{*} \bigr\Vert ^{2} \\ &{}+(1-\beta_{k}-\gamma_{k})\bigl[(1-\lambda_{k} \tau) \bigl\Vert x_{k}-x^{*} \bigr\Vert ^{2}+2\lambda _{k}\bigl\langle F\bigl(x^{*}\bigr),j\bigl(x^{*}-v_{k}\bigr)\bigr\rangle \bigr] \\ =&\bigl[1-\lambda_{k}(1-\beta_{k}-\gamma_{k})\tau \bigr] \bigl\Vert x_{k}-x^{*} \bigr\Vert ^{2} \\ &{}+\lambda_{k}(1-\beta_{k}-\gamma_{k})\tau\cdot \frac{2}{\tau}\bigl\langle F\bigl(x^{*}\bigr),j\bigl(x^{*}-v_{k}\bigr) \bigr\rangle . \end{aligned}$$

Since $\sum^{\infty}_{k=1}\lambda_{k}=\infty$, $\limsup_{k\to\infty}(\beta _{k}+\gamma_{k})<1$ and $\tau=1-\sqrt{\frac{1-\delta}{\zeta}}\in(0,1)$, we get

$$\sum^{\infty}_{k=1}\lambda_{k}(1- \beta_{k}-\gamma_{k})\tau=\infty. $$

Taking into account $\limsup_{k\to\infty}\langle F(x^{*}),j(x^{*}-v_{k})\rangle\leq0$, we can apply Lemma 13 to relation (56) and conclude that $x_{k}\to x^{*}$ as $k\to\infty$. □

## References

[1] Cioranescu I (1990). Geometry of Banach Spaces, Duality Mappings and Nonlinear Problems.

[2] Yao Y, Liou YC, Kang SM, Yu Y (2011). Algorithms with strong convergence for a system of nonlinear variational inequalities in Banach spaces. Nonlinear Anal..

[3] Ceng LC, Gupta H, Ansari QH (2015). Implicit and explicit algorithms for a system of nonlinear variational inequalities in Banach spaces. J. Nonlinear Convex Anal..

[4] Ceng LC, Wang CY, Yao JC (2008). Strong convergence theorems by a related extragradient method for a general system of variational inequalities. Math. Methods Oper. Res..

[5] Verma RU (1999). On a new system of nonlinear variational inequalities and associated iterative algorithms. Math. Sci. Res. Hot-Line.

[6] Verma RU (2001). Projection methods, algorithms and a new system of nonlinear variational inequalities. Comput. Math. Appl..

[7] Facchinei F, Pang JS (2003). Finite-Dimensional Variational Inequalities and Complementarity Problems I.

[8] Glowinski R, Lions JL, Tremolieres R (1981). Numerical Analysis and Variational Inequalities.

[9] Yao YH, Chen R, Xu HK (2010). Schemes for finding minimum-norm solutions of variational inequalities. Nonlinear Anal..

[10] Yao YH, Liou YC, Kang SM (2010). Approach to common elements of variational inequality problems and fixed point problems via a relaxed extragradient method. Comput. Math. Appl..

[11] Yao YH, Noor MA, Liou YC, Kang SM (2012). Iterative algorithms for general multivalued variational inequalities. Abstr. Appl. Anal..

[12] Zegeye H, Shahzad N, Yao YH (2015). Minimum-norm solution of variational inequality and fixed point problem in Banach spaces. Optimization.

[13] Korpelevich GM (1976). An extragradient method for finding saddle points and for other problems. Èkon. Mat. Metody.

[14] Yao YH, Noor MA, Liou YC (2012). Strong convergence of a modified extragradient method to the minimum-norm solution of variational inequalities. Abstr. Appl. Anal..

[15] Yao YH, Postolache M, Liou YC, Yao Z (2016). Construction algorithms for a class of monotone variational inequalities. Optim. Lett..

[16] Aoyama K, Iiduka H, Takahashi W (2006). Weak convergence of an iterative sequence for accretive operators in Banach spaces. Fixed Point Theory Appl..

[17] Yamada I, Batnariu D, Censor Y, Reich S (2001). The hybrid steepest-descent method for the variational inequality problems over the intersection of the fixed-point sets of nonexpansive mappings. Inherently Parallel Algorithms in Feasibility and Optimization and Their Applications.

[18] Buong N, Phuong NTH (2013). Strong convergence to solutions for a class of variational inequalities in Banach spaces by implicit iteration methods. J. Optim. Theory Appl..

[19] Zeng LC, Yao JC (2006). Implicit iteration scheme with perturbed mapping for common fixed points of a finite family of nonexpansive mappings. Nonlinear Anal..

[20] Ceng LC, Petrusel A, Yao JC (2007). Implicit iteration scheme with perturbed mapping for common fixed points of a finite family of Lipschitz pseudocontractive mappings. J. Math. Inequal..

[21] Buong N, Anh NTQ (2011). An implicit iteration method for variational inequalities over the set of common fixed points of a finite family of nonexpansive mappings in Hilbert spaces. Fixed Point Theory Appl..

[22] Kamimura S, Takahashi W (2002). Strong convergence of a proximal-type algorithm in a Banach space. SIAM J. Optim..

[23] Goebel K, Reich S (1984). Uniform Convexity, Hyperbolic Geometry, and Nonexpansive Mappings.

[24] Takahashi Y, Hashimoto K, Kato M (2002). On sharp uniform convexity, smoothness, and strong type, cotype inequalities. J. Nonlinear Convex Anal..

[25] Xu HK (1991). Inequalities in Banach spaces with applications. Nonlinear Anal..

[26] Reich S (1979). Weak convergence theorems for nonexpansive mappings in Banach spaces. J. Math. Anal. Appl..

[27] Yao YH, Liou YC, Kang SM (2013). Two-step projection methods for a system of variational inequality problems in Banach spaces. J. Glob. Optim..

[28] Zeidler E (1985). Nonlinear Functional Analysis and Its Applications III: Variational Methods and Applications.

[29] Ceng LC, Ansari QH, Yao JC (2008). Mann-type steepest-descent and modified steepest-descent methods for variational inequalities in Banach spaces. Numer. Funct. Anal. Optim..

[30] Takahashi W (1997). Weak and strong convergence theorems for families of nonexpansive mappings and their applications. Ann. Univ. Mariae Curie-Skłodowska, Sect. A.

[31] Kikkawa M, Takahashi W (2008). Strong convergence theorems by the viscosity approximation methods for a countable family of nonexpansive mappings. Taiwan. J. Math..

[32] Browder FE (1965). Nonexpansive nonlinear operators in a Banach space. Proc. Natl. Acad. Sci. USA.

[33] Suzuki T (2005). Strong convergence of Krasnoselskii and Mann’s type sequences for one-parameter nonexpansive semigroups without Bochner integrals. J. Math. Anal. Appl..

[34] Xu HK (2002). Iterative algorithms for nonlinear operators. J. Lond. Math. Soc..

